# Diagnostic Accuracy of Machine Learning-Enhanced Methods for Pulmonary Hypertension: A Systematic Review and Meta-Analysis

**DOI:** 10.3390/diagnostics16193106

**Published:** 2026-09-24

**Authors:** Faizan Ahmed, Ayesha Zulfiqar, Tushar Kanti Bhadra, Noor Ul Sabah, Taha Alam, Unaiza Iftikhar, Ahmad Zulaid, Hassan Farooq, Qais Bin Abdul Ghaffar, Muhammad Faizan Tahir, Muhammad Haris, Omar Kamel, Haris Bin Tahir, Gabriel Benjamen, Ali Abbas Khaleel, Ali Ghani, Amro Taha, Fawaz Alenezi

**Affiliations:** 1 Department of Internal Medicine, Jersey Shore University Medical Center, Neptune, NJ 07753, USA; 2 Dow Medical College, Dow University of Health Sciences, Karachi 74200, Pakistan; 3 National Institute of Cardiovascular Diseases, Dhaka 1207, Bangladesh; drtushar106@gmail.com; 4 Quaid-E-Azam Medical College, University of Health Sciences, Bahawalpur 63100, Pakistanahmadzulaid@gmail.com (A.Z.); dr.haris251@gmail.com (M.H.); 5 Ameer-Ud-Din Medical College, University of Health Sciences (UHS), Lahore 54000, Pakistan; tahaaalam9@gmail.com (T.A.); hassanfarooqijaz@gmail.com (H.F.); 6 Foundation University Medical College, Foundation University Islamabad, Islamabad 44000, Pakistan; unaizaiftikhar648@gmail.com; 7 Department of Internal Medicine, Camden Clark Medical Center, WVU Medicine, Parkersburg, WV 26101, USA; 8 Shalamar Medical and Dental College, University of Health Sciences, Lahore 54840, Pakistan; faizantahir5678@gmail.com; 9 Department of Physical Therapy, Qena University, Qena 83523, Egypt; 10 Department of Internal Medicine, Lahore General Hospital, Lahore 54000, Pakistan; harristahirchh@gmail.com; 11 School of Medicine, St. George’s University, St. George’s P.O. Box 7, Grenada; 12 College of Medicine, Al-Mustansiriyah University, Baghdad 14022, Iraq; dr.ali.abbas.k@gmail.com; 13 Department of Cardiology, University of Arizona, Tucson, AZ 85724, USA; alighani152@gmail.com; 14 Division of Cardiology, West Virginia University, Morgantown, WV 26506, USA; taha61591@gmail.com; 15 Division of Cardiology, Department of Medicine, Duke University School of Medicine, Durham, NC 27710, USA; fawaz.enezi@sgu.edu

**Keywords:** pulmonary hypertension, machine learning, deep learning, diagnostic accuracy, QUADAS-2, Reitsma bivariate model, GRADE, meta-analysis

## Abstract

**Background/Objectives:** Right heart catheterization (RHC) is the diagnostic gold standard for pulmonary hypertension (PH), defined as mean pulmonary arterial pressure (mPAP) greater than 20 mmHg at rest. It is invasive and carries procedural risk. Machine learning (ML) and deep learning (DL) applied to non-invasive modalities may provide a non-invasive adjunct for screening, triage, and earlier identification. **Methods:** Methodological quality was assessed using QUADAS-2. Primary non-comparative pooling was restricted to RHC-confirmed studies using Reitsma hierarchical bivariate random-effects models. Comparative studies were analyzed using a paired hierarchical bivariate model, with between-method differences assessed using paired Wald tests. Sensitivity, specificity, likelihood ratios, and diagnostic odds ratios were estimated; predictive values were evaluated as secondary outcomes. GRADE certainty was assessed separately for non-comparative ML/DL, comparative ML/DL, and traditional methods. **Results:** Nine studies involving multiple pulmonary hypertension subtypes met the inclusion criteria: five non-comparative (single-arm) studies including approximately 26,530 participants and four comparative (double-arm) studies including 330 paired participants per arm. In the primary synthesis restricted to RHC-confirmed single-arm studies, ML/DL models achieved a pooled sensitivity of 0.720 (95% CI: 0.647–0.783) and specificity of 0.883 (95% CI: 0.796–0.936), with a diagnostic odds ratio of 19.4 (95% CI: 13.6–27.7). Estimates were similar when all five single-arm studies were included (sensitivity 0.716, 95% CI: 0.654–0.771; specificity 0.891, 95% CI: 0.820–0.937). In comparative studies analyzed with a paired hierarchical bivariate model, ML/DL outperformed traditional methods for both sensitivity (0.838, 95% CI: 0.668–0.930 versus 0.707, 95% CI: 0.421–0.889; paired Wald *p* = 0.019) and specificity (0.849, 95% CI: 0.512–0.968 versus 0.640, 95% CI: 0.376–0.840; paired Wald *p* = 0.0076). At a 25% pre-test probability, ML/DL was projected to yield 34 additional true positives (95% CI: −30 to 109) and 142 fewer false positives (95% CI: −381 to 129) per 1000 patients tested, although all absolute difference intervals crossed zero. Certainty of evidence was very low across all outcomes. GRADE certainty ranged from low to very low depending on outcome and stratum. **Conclusions:** ML/DL-based methods showed promising non-invasive diagnostic accuracy for PH, with significantly higher pooled sensitivity and specificity than traditional methods in paired comparisons. However, evidence certainty ranged from low to very low, and projected absolute benefits remained imprecise. These findings are hypothesis-generating; ML/DL tools may serve as adjuncts in the diagnostic pathway but cannot replace RHC at this stage.

## 1. Introduction

Pulmonary hypertension (PH) is defined as a mean pulmonary arterial pressure (mPAP) greater than 20 mmHg at rest, as measured by right heart catheterization (RHC). PH is classified into five clinical groups according to shared pathophysiological mechanisms, clinical characteristics, hemodynamic profiles, and therapeutic approaches [1]. Its clinical presentation is often non-specific: dyspnea, fatigue, and reduced exercise tolerance overlap substantially with other cardiorespiratory conditions, and diagnostic delay frequently exceeds one year from symptom onset to confirmed diagnosis [2]. Despite advances in treatment, three-year mortality in pulmonary arterial hypertension remains approximately 21% [3].

The diagnostic reference standard is RHC, which provides direct hemodynamic assessment and differentiates pre-capillary from post-capillary disease [4]. However, its invasive nature limits its suitability as a routine first-line investigation in patients presenting with nonspecific symptoms. A less invasive, accessible adjunct tool that identifies patients warranting RHC referral would meaningfully shorten the diagnostic pathway and reduce avoidable disease progression.

Machine learning (ML) encompasses computational systems that derive predictive models directly from data. Artificial intelligence (AI) denotes the overarching field; ML is a subset in which algorithms learn from data rather than explicit programming; deep learning (DL) is a further subset based on multi-layer artificial neural networks. Topol has highlighted that such systems are achieving or approaching clinician-level performance across multiple diagnostic imaging tasks, motivating their evaluation as frontline or adjunctive decision-support tools [5]. In the context of PH, echocardiography-based ML models have demonstrated AUC values exceeding 0.83 against an RHC reference standard [6]. Multimodal DL approaches spanning ECG, chest radiograph, and CT imaging have shown consistently positive discrimination across different patient populations [7].

A prior systematic review restricted to pulmonary arterial hypertension (WHO Group 1) reported pooled ML sensitivity and specificity of approximately 81% and 84%, respectively [8]. However, previous evidence remained limited in clinical scope and did not distinguish non-comparative studies from head-to-head comparisons with conventional diagnostic methods. The present meta-analysis addresses these gaps by including multiple PH subtypes across WHO Groups 1–4 and neonatal PH, analysing single- and double-arm studies separately, applying hierarchical bivariate models, and directly comparing ML/DL with traditional diagnostic approaches. We therefore aimed to evaluate the diagnostic accuracy and comparative performance of ML/DL-based methods for PH detection.

## 2. Methods

This study followed PRISMA 2020 guidelines [9] and the PRISMA extension for diagnostic test accuracy reviews (PRISMA-DTA) and was prospectively registered with PROSPERO (CRD420251131645). No deviations from the registered PROSPERO protocol occurred during the conduct of this review (Supplementary Table S1).

### 2.1. Search Strategy

A systematic search was conducted across PubMed, Embase, Scopus, ScienceDirect, and the Cochrane Library from inception through October 2025. Search terms combined keywords related to pulmonary hypertension (“pulmonary hypertension,” “pulmonary arterial hypertension,” “PAH,” and “CTEPH”), artificial intelligence (“artificial intelligence,” “machine learning,” “deep learning,” and “neural networks”), and diagnostic methods (“diagnosis,” “screening,” “echocardiography,” “electrocardiography,” “chest radiography,” “computed tomography,” and “imaging”). Boolean operators AND and OR were used, and the search strategy was adapted for each database. Duplicate records identified across databases were removed using Rayyan AI prior to title and abstract screening. Detailed search strategies are provided in (Supplementary Table S2). Full-text exclusion reasons for all 128 excluded records, along with their mapping to the four broad PRISMA categories, are provided in Supplementary Table S6.

### 2.2. Eligibility Criteria

Studies were included if they enrolled patients of any age, including pediatric and neonatal populations, with PH or a PH subtype as the target condition. Neonatal studies were retained as a clinically distinct population in which RHC is often impractical or not routinely performed; validated echocardiographic criteria were therefore accepted as the reference standard in this population. An ML or DL algorithm must have been used as the diagnostic index test applied to any imaging, electrocardiographic, echocardiographic, physiological, clinical, or multimodal data. RHC (mPAP > 20 mmHg) or validated echocardiographic thresholds were required as the reference standard. At least one extractable diagnostic accuracy statistic must have been reported. Both non-comparative (single-arm) diagnostic-accuracy studies and head-to-head (double-arm) studies comparing ML/DL with traditional diagnostic methods were eligible. Both retrospective and prospective designs were accepted. Excluded studies were non-English publications, case reports and case series; reviews, editorials, conference abstracts, animal studies, and studies where the ML/DL model did not yield a direct PH diagnostic output. The PIRD framework and detailed eligibility criteria are provided in the Supplemental S2.3, S2.4.1 and S2.4.2.

### 2.3. Data Extraction

Data were extracted independently by four reviewers using a standardized form, including study design, country, sample size, patient age, imaging modality, ML model architecture, reference standard, and all diagnostic accuracy statistics. Study-level diagnostic information is summarized in Supplementary Table S5. Discrepancies were resolved by consensus. For each study, TP, FP, FN, and TN were recorded when directly reported or when the published information allowed a unique integer reconstruction. Comparative-study 2 × 2 counts are presented in Supplementary Table S7. When a study reported more than one candidate model or threshold, we extracted the result its authors identified as their primary or best-performing option, reflecting the result the authors themselves considered most representative of the tool’s diagnostic performance. This approach, while consistent across studies, does not fully eliminate the potential for optimism bias since that ‘best’ result may itself have been chosen based on test performance.

### 2.4. Risk of Bias Assessment

Methodological quality was assessed using QUADAS-2 [10] across four domains: patient selection (D1), index test (D2), reference standard (D3), and flow and timing (D4). Two reviewers assessed each study independently; disagreements were resolved through discussion. Risk-of-bias assessments were visualized using robvis [11] (Supplementary Figure S1). We added three signalling questions to Domains 1 and 2 of QUADAS-2 to capture bias sources specific to AI/ML studies. One question was added to Domain 1: whether train/test splitting was performed at the patient level rather than the image/scan level, preventing the same patient’s data from appearing in both sets. Two questions were added to Domain 2: whether the classification/regression threshold or hyperparameters were prespecified rather than optimized using test-set performance, and whether there was evidence of data leakage (e.g., feature selection, normalization, or dimensionality reduction performed on the full dataset before the train/test split). No new signalling questions were added to Domain 3 or Domain 4. For Domain 3 specifically, the clinical difficulty of performing a reference-standard test was considered separately from risk of bias. Difficulty obtaining a reference standard did not itself justify a low-risk rating; potential misclassification from using an alternative standard was assessed independently.

### 2.5. Statistical Analysis

Primary non-comparative analyses used a Reitsma hierarchical bivariate random-effects model (mada package, R 4.3), jointly modeling logit-transformed sensitivity and specificity; PLR, NLR, and DOR were derived from the fitted model. Only arm-specific estimates are reported for double-arm studies (no composite cross-arm estimate). Single-arm primary synthesis was restricted to RHC-confirmed studies; the one non-RHC-referenced study was excluded from primary pooling, reported separately, and included in the all-study sensitivity analysis. Single-arm and double-arm studies were analyzed in separate strata, with additional analyses by intended clinical task (general PH detection, subtype differentiation, RHC-referral screening, neonatal PH), as these are distinct diagnostic questions. Single-arm task strata with >1 study were pooled; double-arm task strata (1–2 studies each) were reported at the study level. Diagnostic performance was taken preferentially from held-out validation cohorts. Validation procedures were summarized descriptively, distinguishing independent external validation from internal holdout testing and cross-validation. No pooled comparison by validation design is presented. 2 × 2 cells used directly reported counts where available; ambiguous rounding was marked as not uniquely reconstructable rather than estimated, zero cells were retained as zero (with 0.5 continuity correction applied during modeling, Swinnen 2023) [12], and missing metrics were recorded as not reported. Study-level diagnostic information and comparative 2 × 2 counts are presented in Supplementary Tables S5 and S7, respectively. For double-arm studies, because ML/DL and traditional methods were evaluated in the same patients, the two arms were analyzed jointly within a paired hierarchical bivariate model, with between-arm differences tested via a paired Wald test (10^−4^ boundary clamp; convergence and parameters in Supplementary Table S4). Leave-one-out analyses assessed the influence of individual studies on the pooled DOR in the five-study non-comparative synthesis (Supplementary Figure S12) and the paired comparative synthesis. Heterogeneity was characterized via tau^2^; I^2^ for secondary outcomes was obtained from supplementary proportional analyses. PPV/NPV were pooled separately (random-effects, logit transformation) given prevalence-dependence. Publication bias testing was not performed (fewer than 10 studies per stratum). Projected absolute impact was calculated at pre-test probabilities of 25%, 50%, and 75%, representing an enriched screening population, an intermediate-suspicion referral population, and a high-suspicion pre-RHC population, respectively.

### 2.6. GRADE Assessment

Certainty of evidence was assessed using the GRADE approach for diagnostic test accuracy across all three analytical strata independently: (1) single-arm DL models, (2) double-arm ML/DL models, and (3) double-arm traditional methods. Both true-positive rate (sensitivity) and true-negative rate (specificity) outcomes were evaluated across five GRADE domains: risk of bias, indirectness, inconsistency, imprecision, and publication bias. Effects were expressed as numbers of true positives, false negatives, true negatives, and false positives per 1000 patients tested at three pre-test probabilities (25%, 50%, 75%, Supplementary Table S3).

## 3. Results

### 3.1. Study Selection

Database searches across five sources identified 3203 records. After removing 1679 duplicates, 1524 records were screened, and 1387 were excluded. 137 full-text reports were assessed for eligibility, of which 128 were excluded across four prespecified categories (wrong study design; wrong outcomes; insufficient data for 2 × 2 reconstruction; no ML/DL model), with study-level reasons in Supplementary Table S5. Nine studies met the inclusion criteria and were included in the final review (Figure 1).

### 3.2. Study Characteristics and Intended Clinical Task

Five single-arm (non-comparative) studies evaluated ML/DL models in isolation (Liu et al. 2024 [13]; Salehi et al. 2025 [14]; Shirahata et al. 2025 [15]; Swinnen et al. 2023 [12]; Ragnarsdóttir et al. 2024 [16]; approximately 26,530 participants), and four double-arm (comparative) studies evaluated ML/DL and traditional methods in the same patients (Hirata et al. 2024 [17]; Imai et al. 2024 [18]; Argiento et al. 2024 [19]; Xie et al. 2025 [20]; 330 paired participants). Study characteristics are summarized in Table 1. Each model’s intended clinical task was classified a priori as PH detection (general), PH subtype differentiation, RHC-referral screening, or neonatal PH detection, and additional analyses were organized by clinical task. Within the comparative set, the intended tasks were PH detection (general) for Imai 2024 (chest radiography) [18] and Xie 2025 [20] (CT pulmonary angiography), PH subtype differentiation for Hirata 2024 (echocardiography), and RHC-referral screening for Argiento 2024 (anamnestic data plus transthoracic echocardiography) (Supplementary Table S7). Validation designs varied substantially. Liu et al. [13] used separate development, tuning, internal-validation, and external-validation cohorts; Salehi et al. [14] evaluated an automated echocardiographic measurement tool against RHC without an external validation cohort; Shirahata et al. [15] used a 70:30 stratified train/test split with 10-fold cross-validation; Swinnen et al. [12] reported both an internal test cohort and an independent external validation cohort; Ragnarsdóttir et al. [16] used patient-level cross-validation with an internal held-out test cohort; Hirata et al. [17] used a 720-patient derivation cohort with stratified 10-fold cross-validation and a separate prospective 165-patient validation cohort; Imai et al. [18] used a development/test split with four-fold cross-validation; Argiento et al. used a 60% training/validation and 40% held-out test split with repeated 3-fold cross-validation; and Xie et al. [20] used an 8:2 training/test split with repeated 10-fold cross-validation. Study-level validation designs, thresholds and overfitting safeguards are detailed in Table 1. Diagnostic information reported in the included studies is summarized in Supplementary Table S5, and comparative-study 2 × 2 counts are presented in Supplementary Table S7.

### 3.3. Risk of Bias

QUADAS-2 results, incorporating three additional AI-specific signaling questions in Domains 1 and 2 (patient-level vs. image-level data separation, data leakage between development and test sets, and threshold selection in the test set), are shown in Supplementary Figure S1. Salehi 2025 was rated low risk across all domains [14]. Shirahata 2025 [15], Ragnarsdóttir 2024 [16], and Imai 2024 were rated high overall risk [18], driven by patient-selection concerns (D1); Liu 2024 was rated high overall risk owing to reference-standard concerns (D3) [13]. Ragnarsdóttir 2024 was additionally rated high risk in D3 because echocardiographic rather than RHC-based criteria were used; the clinical impracticality of RHC in neonates was treated as an applicability consideration and did not offset the methodological misclassification risk [16]. Xie 2025 [20], Swinnen 2023 [12], Argiento 2024 [19], and Hirata 2024 [17] were rated as having some concerns. These findings contributed to serious risk-of-bias downgrading in all GRADE assessments (Supplementary Table S3).

### 3.4. Primary Single-Arm Analysis: RHC-Confirmed Studies

Consistent with the prespecified primary synthesis, single-arm pooling was restricted to studies in which PH status was confirmed by right heart catheterization (Liu 2024 [13]; Salehi 2025 [14]; Shirahata 2025 [15]; Swinnen 2023 [12]). The Reitsma bivariate model converged, with a 0.5 continuity correction for one zero cell (Swinnen 2023 [12]). Pooled sensitivity was 0.720 (95% CI: 0.647–0.783) and pooled specificity 0.883 (95% CI: 0.796–0.936), with a diagnostic odds ratio of 19.4 (95% CI: 13.6–27.7), positive likelihood ratio 6.16 (95% CI: 3.81–10.2), and negative likelihood ratio 0.318 (95% CI: 0.27–0.378) (Figure 2). The single study using a non-invasive reference standard (Ragnarsdóttir 2024 [16], echocardiographic criteria in neonates) was not pooled with RHC-confirmed studies and is summarized narratively in Section 3.6 (sensitivity 0.771, 95% CI: 0.573–0.894; specificity 0.938, 95% CI: 0.842–0.977) Figure 2 and Figure 3.

### 3.5. Sensitivity Analysis: All Single-Arm Studies

Including all five single-arm studies irrespective of reference standard yielded nearly identical estimates: pooled sensitivity 0.716 (95% CI: 0.654–0.771), specificity 0.891 (95% CI: 0.820–0.937), DOR 20.7 (95% CI: 14.8–29.1), PLR 6.61 (95% CI: 4.24–10.5), and NLR 0.319 (95% CI: 0.276–0.371) (Supplementary Figures S2–S6), indicating that inclusion of the non-RHC reference study did not materially alter the pooled results.

### 3.6. Single-Arm Synthesis Stratified by Clinical Task

For PH detection (general), two studies contributed (Liu 2024 [13]; Salehi 2025 [14]): pooled sensitivity 0.732 (95% CI: 0.603–0.831) and specificity 0.869 (95% CI: 0.686–0.953) (Supplementary Figures S7 and S8). All remaining task strata contained a single study and were synthesized narratively rather than pooled: PAH versus PH-LHD discrimination (Swinnen 2023 [12]; sensitivity 0.696, 95% CI: 0.560–0.805; specificity 0.991, 95% CI: 0.919–0.999), CT-based PAH detection (Shirahata 2025 [15]; sensitivity 0.729, 95% CI: 0.529–0.866; specificity 0.870, 95% CI: 0.697–0.951), and neonatal PH detection against echocardiographic criteria (Ragnarsdóttir 2024 [16]; sensitivity 0.771, 95% CI: 0.573–0.894; specificity 0.938, 95% CI: 0.842–0.977). No composite estimate pooling across disparate tasks or modalities is reported.

### 3.7. Validation Procedures

Validation procedures varied across studies. Liu 2024 [13] and Swinnen 2023 [12] reported independent validation cohorts, whereas Ragnarsdóttir 2024 [16] used a patient-level held-out test set from a single source cohort, representing internal holdout testing rather than external validation. Because the previous validation-design subgroup combined distinct validation procedures, its pooled estimates have been removed. Validation characteristics are summarized descriptively in Table 1.

### 3.8. Comparative (Double-Arm) Studies: Paired Hierarchical Bivariate Analysis

In four double-arm studies (330 paired participants), ML/DL and traditional methods were evaluated in the same patients; accordingly, the two arms were analyzed jointly within a paired hierarchical bivariate model with formal paired significance testing. Both model components converged, with a 10^−4^ boundary clamp to prevent degenerate proportion estimates (Supplementary Table S4). ML/DL achieved pooled sensitivity 0.838 (95% CI: 0.668–0.930) versus 0.707 (95% CI: 0.421–0.889) for traditional methods (paired Wald *p* = 0.019), and pooled specificity 0.849 (95% CI: 0.512–0.968) versus 0.640 (95% CI: 0.376–0.840) (paired Wald *p* = 0.0076) (Figure 4 and Figure 5). Corresponding pooled DORs were 29.3 (95% CI: 4.16–201) for ML/DL versus 4.27 (95% CI: 0.86–21.6) for traditional methods, with PLR 5.54 (95% CI: 1.66–26.4) versus 1.93 (95% CI: 0.932–4.52) and NLR 0.20 (95% CI: 0.0824–0.453) versus 0.471 (95% CI: 0.166–1.08) (Supplementary Figures S9–S11). Only arm-specific estimates are reported; no composite estimate pooling both index tests is presented.

### 3.9. Stratified Comparative Analyses (Modality, Clinical Task, Age)

Because each modality–task stratum contained one or two studies, comparative strata were synthesized narratively at the study level (per-study 2 × 2 counts in Supplementary Table S7). Within PH detection (general), ML/DL specificity exceeded the paired traditional arm in Imai 2024 (chest radiography: 0.986 vs. 0.901) and Xie 2025 (CTPA: 0.929 vs. 0.429), with identical sensitivity in Xie 2025 (0.909 in both arms) and higher ML/DL sensitivity in Imai 2024 (0.933 vs. 0.833) [18,20]. In the single PH-subtype-differentiation study (Hirata 2024 [17], echocardiography) and the single RHC-referral-screening study (Argiento 2024 [19]), ML/DL exceeded the traditional comparator on both sensitivity and specificity, with overlapping per-study confidence intervals throughout. Age-stratified paired models were fitted by cohort median age; model parameters are reported in Supplementary Table S4. In younger cohorts (Imai 2024 [18], Xie 2025 [20]), traditional and ML/DL sensitivity were similar (0.877, 95% CI: 0.777–0.936 vs. 0.918, 95% CI: 0.835–0.961), while ML/DL specificity was numerically higher (0.968, 95% CI: 0.782–0.996 vs. 0.728, 95% CI: 0.187–0.969). In older cohorts (Hirata 2024 [17], Argiento 2024 [19]), ML/DL was numerically higher for both sensitivity (0.722, 95% CI: 0.500–0.871 vs. 0.477, 95% CI: 0.357–0.600) and specificity (0.671, 95% CI: 0.560–0.765 vs. 0.506, 95% CI: 0.397–0.615). With two studies per band, these estimates are exploratory.

### 3.10. Projected Impact per 1000 Patients Tested (Paired Model)

Absolute projected effects were calculated from the paired-model estimates with 95% confidence intervals at pre-test probabilities of 25%, 50%, and 75%, representing an enriched screening population, a referral population with intermediate suspicion, and a pre-RHC population with high suspicion, respectively (justification in Section 2.5). At 25% pre-test probability, ML/DL was projected to yield 34 additional true positives (95% CI: −30 to 109) and 142 fewer false positives (95% CI: −381 to 129) per 1000 patients tested relative to traditional methods; corresponding differences were 68 additional true positives (95% CI: −60 to 218) and 95 fewer false positives (95% CI: −254 to 86) at 50%, and 102 additional true positives (95% CI: −90 to 327) and 47 fewer false positives (95% CI: −127 to 43) at 75% (Table 2). Although the paired between-arm comparisons of sensitivity and specificity reached statistical significance on the logit scale (Section 3.8), all absolute difference intervals per 1000 crossed zero; these projections are therefore imprecise and should be interpreted as exploratory.

### 3.11. Robustness: Leave-One-out and Model Diagnostics

Leave-one-out analyses were performed for both strata. For the five-study single-arm synthesis, exclusion of any individual study yielded pooled sensitivities between 0.676 (excluding Liu 2024 [13]) and 0.751 (excluding Salehi 2025 [14]) and pooled DORs between 19.4 (excluding Ragnarsdóttir 2024 [16]) and 28.6 (excluding Salehi 2025 [14]). All recalculated DOR point estimates fell within the confidence interval of the full-model DOR (20.7, 95% CI: 14.8–29.1) (Supplementary Figure S12). For the paired double-arm model, the ML/DL DOR point estimate exceeded the traditional-arm estimate in every refit; the lowest ML/DL estimate followed exclusion of Imai 2024 [18] (DOR 9.0, 95% CI: 2.8–29.3, vs. 1.9, 95% CI: 0.4–9.4 for the traditional arm) (Figure 6). Model convergence status, logit-scale parameter estimates, standard errors, available correlation parameters, and continuity-correction methods (0.5 zero-cell correction for Swinnen 2023 [12]) in single-arm models; 10^−4^ clamp in paired models) are reported in Supplementary Table S4.

To explore the impact of model selection, we conducted a post-hoc sensitivity analysis using alternative eligible models from studies that reported multiple options. For each such study, we extracted the performance data for the lowest-performing alternative model or threshold that still provided a complete 2 × 2 table. The Reitsma bivariate model was then refitted for the single-arm RHC-confirmed stratum using these conservative estimates. In this conservative scenario, pooled sensitivity was 0.691 (95% CI: 0.622–0.754), specificity was 0.875 (95% CI: 0.790–0.931), and DOR was 16.5 (95% CI: 11.4–23.9). These estimates were numerically lower than the primary estimates (sensitivity 0.720, specificity 0.883, DOR 19.4), with overlapping confidence intervals. The findings suggest lower estimated diagnostic performance when alternative models were selected, although uncertainty regarding model selection bias remains.

### 3.12. Summary of Pooled Estimates

Table 3 summarizes the pooled sensitivity, specificity, diagnostic odds ratio, and likelihood ratios across the non-comparative and paired comparative analyses, with 95% confidence intervals and paired Wald *p* values where applicable.

### 3.13. Publication Bias

Publication bias could not be reliably assessed because of the small number of studies within each analytical stratum. Nevertheless, some concern remains because this represents a small and emerging evidence base.

### 3.14. GRADE Certainty of Evidence

Certainty was rated separately for each stratum and outcome following the GRADE framework for diagnostic test accuracy, with domain-specific justifications and without double-counting single analytical concerns across domains. Risk of bias was rated serious across all six outcomes (high overall QUADAS-2 risk in one or more included studies), as was inconsistency (heterogeneity I^2^ > 50%). Indirectness was rated not serious across all outcomes, as population, index test, and reference standard were consistent within each stratum. Publication bias was undetected, reflecting an insufficient number of studies per stratum for formal assessment rather than a confirmed absence of bias. Imprecision varied by outcome: not serious for single-arm sensitivity, single-arm specificity, and double-arm ML/DL sensitivity (comparatively narrow confidence intervals), and serious for double-arm traditional-method sensitivity, double-arm traditional-method specificity, and double-arm ML/DL specificity (wide confidence intervals). Certainty was therefore low (⊕⊕◯◯) for single-arm sensitivity, single-arm specificity, and double-arm ML/DL sensitivity, and very low (⊕◯◯◯) for double-arm traditional-method sensitivity, double-arm traditional-method specificity, and double-arm ML/DL specificity (Supplementary Table S3).

## 4. Discussion

This meta-analysis synthesizes evidence on ML/DL-enhanced methods for non-invasive PH detection across three analytical strata, with GRADE certainty assessment applied to each. In five non-comparative studies, pooled sensitivity was 0.716 and specificity 0.891. In four head-to-head comparative studies analyzed within a paired hierarchical bivariate model, ML/DL models achieved numerically higher sensitivity (0.838 vs. 0.707) and specificity (0.849 vs. 0.640) than traditional methods, and these between-arm differences reached statistical significance on the logit scale (sensitivity *p* = 0.019; specificity *p* = 0.0076). Corresponding pooled DORs were 29.3 (95% CI: 4.16–201) for ML/DL versus 4.27 (95% CI: 0.86–21.6) for traditional methods, although the DOR difference itself was not statistically significant (*p* = 0.169). GRADE certainty ranged from low to very low across outcomes. All findings remain hypothesis-generating.

### 4.1. Key Findings and Clinical Interpretation

The pooled PLR of 6.16 in the RHC-confirmed single-arm analysis indicates a moderate-to-large shift in post-test probability following a positive result. At an illustrative pre-test probability of 25%, the GRADE table projects 179 true positives and 82 false positives per 1000 tested; these modeled estimates suggest potential utility as a triage or referral-prioritization tool. The NLR of 0.318 indicates a moderate reduction following a negative result but does not reach the threshold of 0.10 typically required to confidently rule out disease. This positions ML/DL models as adjuncts for raising suspicion and guiding RHC referral, rather than definitive diagnostic tests.

The comparative paired analysis provides the most clinically informative framing of the comparative-study results. At 25% pre-test probability, ML/DL models were estimated to identify 34 additional PH patients (95% CI: −30 to 109) and produce 142 fewer false-positive classifications (95% CI: −381 to 129) per 1000 compared with traditional methods. At 50% pre-test probability, the corresponding differences were 68 additional true positives (95% CI: −60 to 218) and 95 fewer false positives (95% CI: −254 to 86); at 75%, 102 additional true positives (95% CI: −90 to 327) and 47 fewer false positives (95% CI: −127 to 43). Although the paired between-arm comparisons of sensitivity and specificity reached statistical significance on the logit scale, all absolute difference intervals per 1000 crossed zero, meaning these projected clinical benefits are imprecise and should be interpreted as exploratory rather than definitive. The direction of effect consistently favored ML/DL, but the magnitude of benefit at the patient level remains uncertain.

### 4.2. Certainty of Evidence

Certainty of evidence ranged from low to very low across the six outcomes, and remains a central interpretive constraint. This reflects downgrading across GRADE domains: serious risk of bias (predominantly retrospective designs with patient selection concerns), not serious indirectness (population, index test, and reference standard were consistent within each stratum), serious inconsistency (high between-study variance), imprecision that was not serious for single-arm sensitivity, single-arm specificity, and double-arm ML/DL sensitivity, but serious for double-arm traditional-method sensitivity, double-arm traditional-method specificity, and double-arm ML/DL specificity (wide confidence intervals from small study samples), and concern regarding publication bias (small emerging field with probable positive-results bias). Low to very low certainty does not mean the findings are incorrect; it means the evidence base does not yet provide sufficient reliability to alter clinical practice or guideline recommendations.

### 4.3. Sources of Heterogeneity

Several structural factors may have contributed to the observed between-study heterogeneity. First, imaging modalities differed substantially; echocardiography, CT/CTPA, chest radiograph, ECG, and multimodal combinations have different diagnostic characteristics. Second, ML architectures ranged from shallow ensemble methods to deep convolutional neural networks. Third, patient populations spanned neonates (Ragnarsdóttir et al. [16]) to adult specialist-referral populations (Salehi et al. [14]). Fourth, reference standards were not uniform, with echocardiographic criteria in the neonatal study introducing a distinct measurement context. Fifth, the traditional-method comparator definition differed across double-arm studies, encompassing guideline-based assessment, clinician clinical judgment, and simple imaging thresholds. Finally, the studies evaluated different clinical questions, including detecting PH, distinguishing between PH subtypes, and identifying patients who may require referral for right heart catheterization.

### 4.4. Comparison with the Existing Literature

Our single-arm pooled sensitivity of 0.716 is somewhat lower than the 81% reported by Fadilah et al. [8], consistent with the more conservative bivariate model and broader scope. Prior work by Anand et al. [5] demonstrated AUC exceeding 0.83 for echocardiography-based ML, consistent with the higher estimates in our double-arm ML arm. The parallel with the cardiac monitoring literature is instructive: CRYSTAL AF showed that extended device-based monitoring detected AF in 12.4% of cryptogenic stroke patients at 12 months versus 2.0% with standard follow-up [21], and STROKESTOP demonstrated that systematic screening with structured downstream management reduced adverse events at six years [22]. These analogies suggest that the clinical value of ML-enhanced PH detection will similarly depend on whether positive results reliably trigger RHC and specialist management. A comparison of the scope, methods, and key features of the previous review and the present meta-analysis is provided in Supplementary Table S8.

### 4.5. Clinical Implications

The evidence supports a potential role for ML/DL as a non-invasive adjunct in the PH diagnostic pathway, specifically for pre-test probability enrichment, which could shorten the diagnostic delay currently exceeding one year in many patients [2]. The GRADE projection of 142 fewer false positives per 1000 at 25% pre-test probability is particularly relevant, as it suggests ML/DL could reduce unnecessary RHC referrals, though the confidence interval crosses zero and the estimate is imprecise. However, the absolute difference intervals all include zero, GRADE certainty is low to very low, and the comparative evidence derives from small, predominantly retrospective single-center studies. Clinical adoption therefore cannot be justified on the current evidence base. Deployment should be conditional on prospective external validation with standardized reference standards and structured care pathways.

### 4.6. Strengths

This is, to our knowledge, among the first meta-analyses to (1) pool traditional-versus-ML/DL comparative diagnostic accuracy under a matched Reitsma bivariate framework with formal paired significance testing; (2) apply GRADE certainty assessment across all three analytical strata independently; and (3) include multiple PH subtypes spanning WHO Groups 1–4, together with a neonatal PH population. All primary metrics were derived from a single bivariate model, ensuring internal consistency. Separate single-arm and double-arm strata prevent conflation of different study designs.

## 5. Limitations

Several limitations must be acknowledged. First, the evidence base was small, with only nine studies and no more than five studies in any analytical stratum, limiting the stability of pooled estimates and the statistical power of comparative analyses. Second, GRADE certainty ranged from low to very low across outcomes and analytical strata. Third, substantial heterogeneity was present because of structural differences in patient populations, target conditions, diagnostic modalities, ML/DL architectures, thresholds, and reference standards, which could not be adequately explored statistically. Some clinical heterogeneity may have arisen from including the neonatal PH study in the all-study sensitivity analysis, and from differences in diagnostic inputs, algorithms, and clinical applications as well. These factors should be considered when interpreting the pooled estimates, which provide a broad overview of AI-assisted diagnostic performance across diverse PH settings rather than age- or modality-specific accuracy. Fourth, the comparative studies had small sample sizes (approximately 330 participants in total), reducing the precision of comparative estimates and this is directly reflected in the wide confidence intervals for the absolute difference projections, all of which crossed zero. Fifth, although the RHC-restricted primary model converged, it included only four studies, limiting the robustness of its pooled estimates. Sixth, meta-regression was not performed because it would be statistically unreliable with five or fewer studies per stratum. Seventh, PPV and NPV were strongly prevalence-dependent and showed substantial heterogeneity (I^2^ > 77%), limiting their generalizability across settings. Eighth, independent external validation was limited. Several studies relied on internal holdout testing or cross-validation, leaving uncertainty about performance in independent populations and settings. Ninth, geographic representation was limited, with little evidence from resource-constrained healthcare settings, thereby limiting the generalizability of the findings. Finally, although we implemented a prespecified selection rule for multi-model studies and conducted a sensitivity analysis using alternative, lower-performing models, the inherent risk of optimistic selection bias in an emerging field like this cannot be completely eliminated. Studies reporting multiple models or thresholds may selectively report the most favorable results, and our selection rule, while systematic, may not fully account for all potential reporting biases. This residual uncertainty should be considered when interpreting the pooled estimates.

## 6. Conclusions

This meta-analysis provides the pooled evidence comparing ML/DL-enhanced and traditional diagnostic accuracy for PH, with GRADE certainty assessment across three analytical strata. ML/DL methods achieved promising performance in non-comparative studies (sensitivity 0.716, specificity 0.891, DOR 20.7) and, in paired comparative analysis, showed statistically significant between-arm differences on the logit scale for both sensitivity (*p* = 0.019) and specificity (*p* = 0.0076), corresponding to an estimated 34 additional true-positive cases (95% CI: −30 to 109) and 142 fewer false-positive classifications (95% CI: −381 to 129) per 1000 individuals tested at a 25% pre-test probability. However, all absolute difference confidence intervals crossed zero, the DOR difference was not statistically significant (*p* = 0.169), and GRADE certainty ranged from low to very low across outcomes. These findings are hypothesis-generating. ML/DL tools may serve as non-invasive adjuncts to facilitate earlier PH referral but cannot replace right heart catheterization at this stage. Prospective external validation with standardized comparative designs is required.

## Figures and Tables

**Figure 1:**
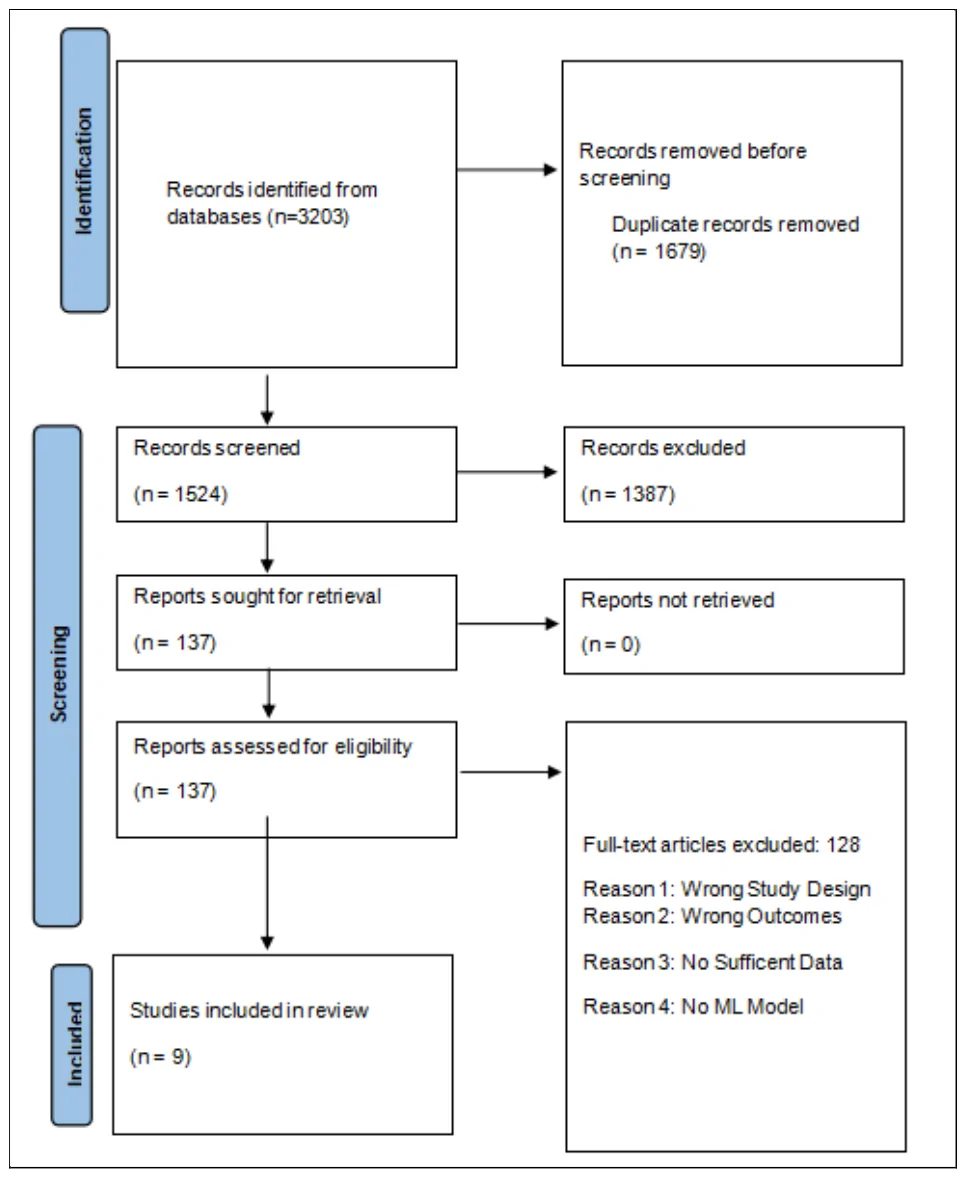
PRISMA 2020 flow diagram. Nine studies were included in the meta-analysis.

**Figure 2:**
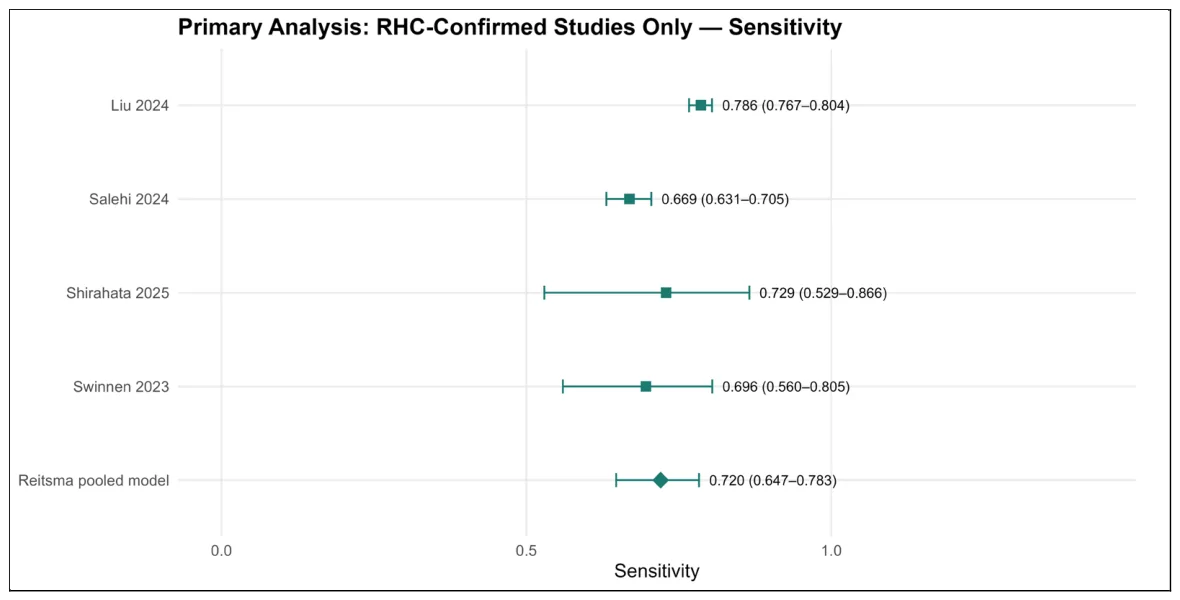
Sensitivity of machine learning/deep learning (ML/DL) models in four RHC-confirmed non-comparative studies [12,13,14,15]. Squares represent study-level estimates, the diamond represents the pooled estimate, and horizontal lines indicate 95% confidence intervals. Pooled sensitivity from the Reitsma hierarchical bivariate model was 0.720 (95% CI: 0.647–0.783). A 0.5 continuity correction was applied during modelling for the zero cell in Swinnen 2023. RHC, right heart catheterization.

**Figure 3:**
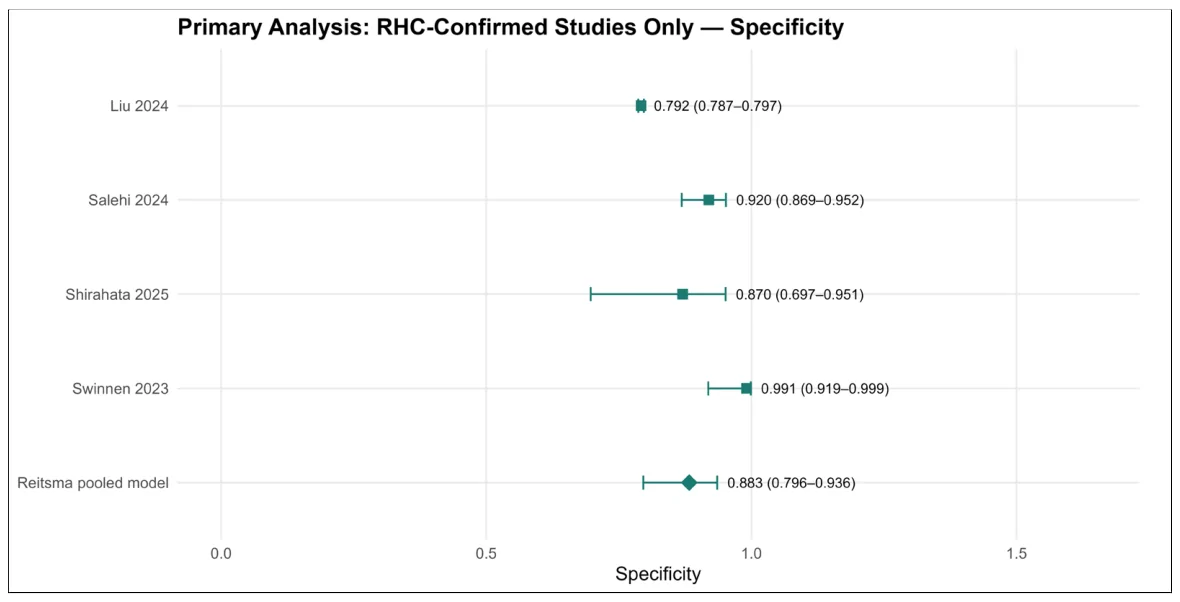
Specificity of machine learning/deep learning (ML/DL) models in four RHC-confirmed non-comparative studies [12,13,14,15]. Squares represent study-level estimates, the diamond represents the pooled estimate, and horizontal lines indicate 95% confidence intervals. Pooled specificity from the Reitsma hierarchical bivariate model was 0.883 (95% CI: 0.796–0.936). A 0.5 continuity correction was applied during modelling for the zero cell in Swinnen 2023. RHC, right heart catheterization.

**Figure 4:**
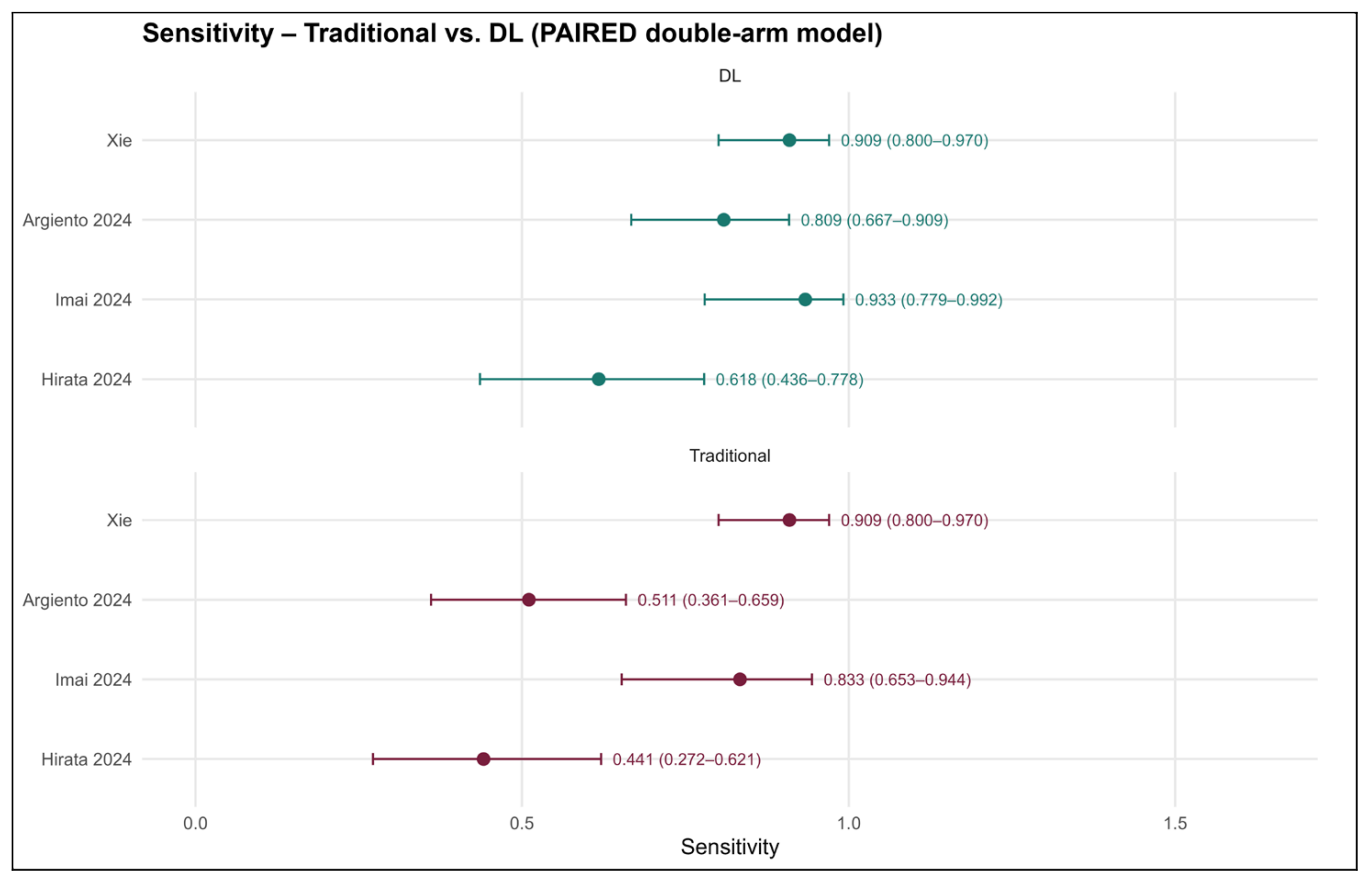
Study-level sensitivity of machine learning/deep learning (ML/DL) models and traditional diagnostic methods in four comparative studies [17,18,19,20]. Teal circles represent ML/DL estimates and burgundy circles represent traditional-method estimates; horizontal lines indicate 95% confidence intervals. The paired hierarchical bivariate model yielded pooled sensitivities of 0.838 (95% CI: 0.668–0.930) for ML/DL and 0.707 (95% CI: 0.421–0.889) for traditional methods (paired Wald *p* = 0.019). Pooled estimates are reported here but are not displayed in the plot.

**Figure 5:**
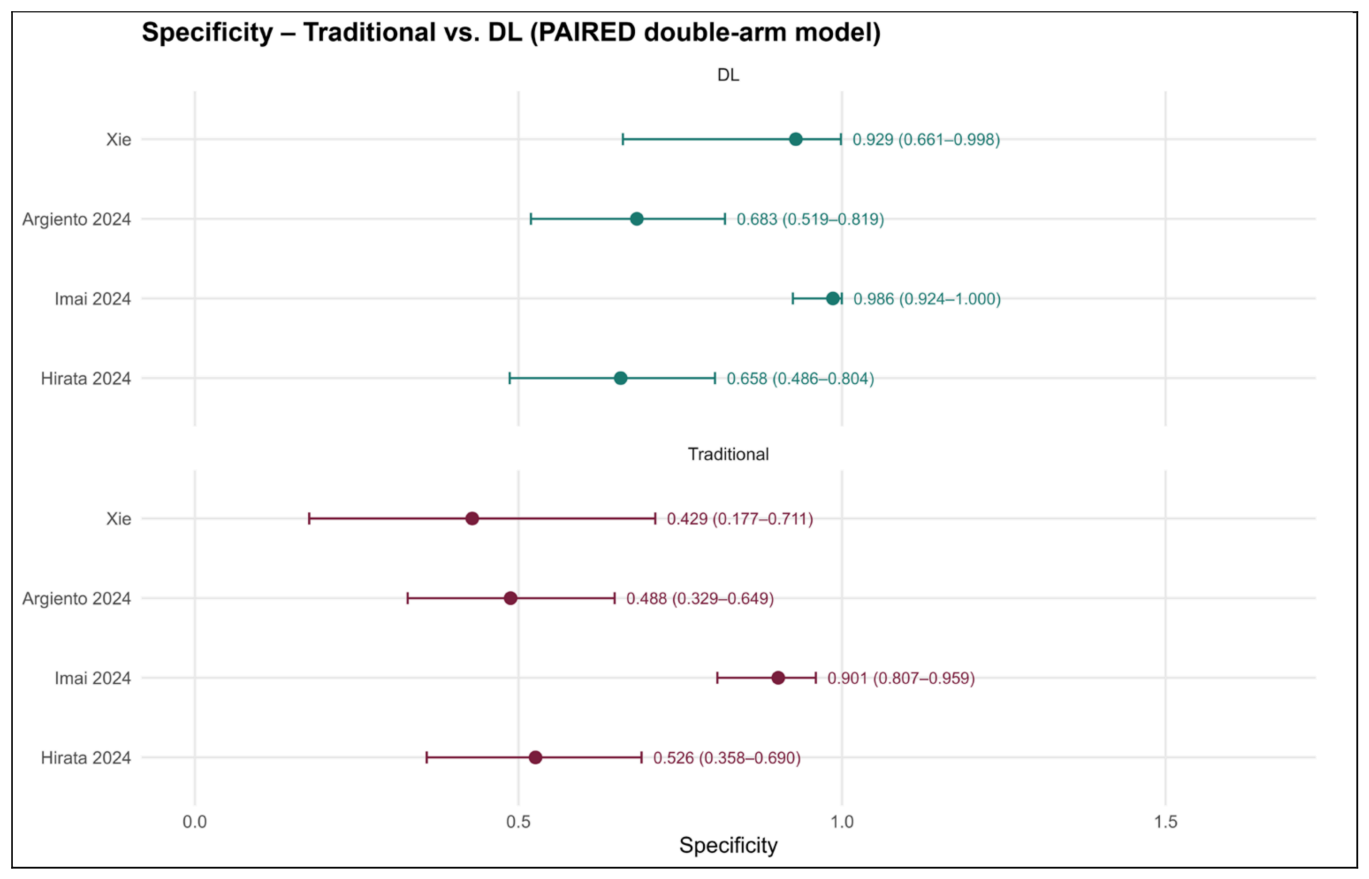
Study-level specificity of machine learning/deep learning (ML/DL) models and traditional diagnostic methods in four comparative studies [17,18,19,20]. Teal circles represent ML/DL estimates and burgundy circles represent traditional-method estimates; horizontal lines indicate 95% confidence intervals. The paired hierarchical bivariate model yielded pooled specificities of 0.849 (95% CI: 0.512–0.968) for ML/DL and 0.640 (95% CI: 0.376–0.840) for traditional methods (paired Wald *p* = 0.0076). Pooled estimates are reported here but are not displayed in the plot.

**Figure 6:**
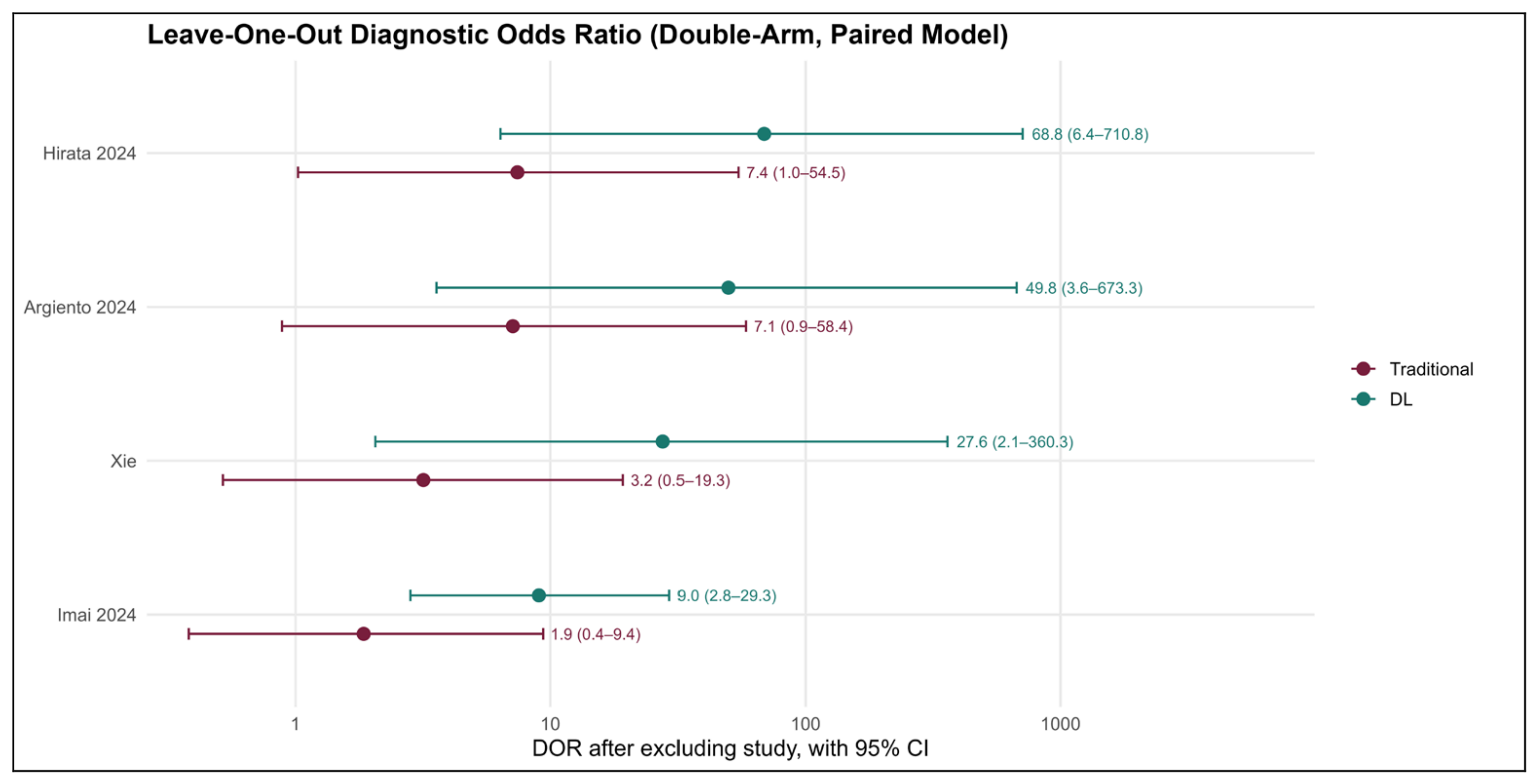
Leave-one-out analysis of the diagnostic odds ratio (DOR) in the paired comparative model [17,18,19,20]. Each row identifies the study omitted before refitting the model. Teal and burgundy circles represent the recalculated pooled DORs for machine learning/deep learning (ML/DL) models and traditional methods, respectively; horizontal lines indicate 95% confidence intervals on a logarithmic scale. The ML/DL point estimate exceeded the traditional-method estimate in every refit. Excluding Imai 2024 yielded the lowest ML/DL DOR of 9.0 (95% CI: 2.8–29.3), compared with 1.9 (95% CI: 0.4–9.4) for traditional methods.

**Table 1:** Characteristics of included studies.

Study	Design/Setting	Modality/Model	PH/Reference Standard	Validation Cohort/Design	Threshold	Overfitting Safeguards
Swinnen et al. [12]	2023; single-arm; Belgium	Echo + ECG + PFT; Random Forest	PAH vs. PH-LHD; RHC	Internal test *n* = 104; external validation *n* = 165 (91 PAH, 74 PH-LHD).	Probability ≥ 0.5.	10-fold CV; grid search; 100 bootstrap models; feature reduction; SHAP.
Liu et al. [13]	2024; single-arm; Taiwan	ECG + CXR; CNN/DenseNet-121 + XGBoost	PAH/CTEPH; RHC (mPAP > 20)	Internal validation *n* = 25,512 + external validation *n* = 16,736; separate development/tuning cohorts.	Youden-index cutoff from tuning set; numeric cutoff NR.	Patient-level separation; oversampling; early stopping; L2 regularization.
Salehi et al. [14]	2025; single-arm; UK	Echocardiography; Ultromics AI	PAH; RHC	787 with automated/manual TRJV and mPAP (620 PH, 167 non-PH); no external validation.	TRJV 3.4 m/s; 2.8 m/s also evaluated.	No imputation; paired automated/manual assessment.
Shirahata et al. [15]	2025; single-arm; Japan	CT; Random Forest using PA volume	PAH; RHC	163; 70% stratified training/30% test; 10-fold CV.	NR.	Stratified split; grid-search optimization; 10-fold CV.
Ragnarsdóttir et al. [16]	2024; single-arm; Iceland	Echocardiography; 3D deep-learning CNN	Neonatal PH; echocardiographic criteria	192 training/validation participants; internal held-out test set of 78 participants from the same source cohort; patient-level splitting.	NR.	Patient-level 10-fold CV; held-out test; pretrained weights; augmentation; weight decay.
Hirata et al. [17]	2024; double-arm; Japan	Echocardiography; elastic-net logistic regression	PH classification; RHC	Derivation *n* = 720 with stratified 10-fold CV + separate prospective validation *n* = 165.	Multiclass; no single binary cutoff.	Elastic-net regularization; stratified 10-fold CV; prospective validation.
Imai et al. [18]	2024; double-arm; Japan	Chest X-ray; ResNet50 DL	PAH; RHC	Test: 87 patients/101 images; 4:1 development/test split; 4-fold CV.	AUC threshold 0.69.	Patient-ID separation; 4-fold CV; augmentation.
Argiento et al. [19]	2024; double-arm; Italy	Echo + clinical history; regularized regression	PAH; RHC	Training *n* = 138 + independent test *n* = 88; repeated 3-fold CV.	Risk-score cutoff 2.155.	Repeated 3-fold CV; grid search; feature limitation; stratified split; synthetic balancing.
Xie et al. [20]	2025; double-arm; China	CTPA; XGBoost radiomic model	PH; RHC	8:2 training/test split; held-out test *n* = 69; no external validation.	Predicted mPAP > 20 mmHg.	5-fold grid search; 20 rounds of 10-fold CV; held-out test.

ECG = electrocardiography; CXR = chest X-ray; CT = computed tomography; PFT = pulmonary function testing; PH-LHD = PH due to left heart disease; RHC = right heart catheterization; CV = cross-validation; NR = not reported. Validation cohort refers to the cohort used for diagnostic performance evaluation.

**Table 2:** Projected true positives and false positives per 1000 patients tested (paired double-arm model; 95% CI).

Pre-Test Probability	TP Traditional	TP ML/DL	FP Traditional	FP ML/DL	ΔTP/ΔFP (ML/DL − Traditional)
25%	173 (105–223)	207 (167–233)	277 (119–470)	135 (24–367)	+34 (−30 to 109)/−142 (−381 to 129)
50%	346 (210–445)	414 (333–465)	185 (79–313)	90 (16–245)	+68 (−60 to 218)/−95 (−254 to 86)
75%	519 (316–668)	620 (500–698)	92 (40–157)	45 (8–122)	+102 (−90 to 327)/−47 (−127 to 43)

TP = true positives; FP = false positives. All between-arm difference intervals include zero.

**Table 3:** Pooled diagnostic accuracy—all analytical strata.

Stratum	Sensitivity (95% CI)	Specificity (95% CI)	DOR (95% CI)	PLR (95% CI)	NLR (95% CI)	Paired Wald *p* (sens/spec)
Single-arm, RHC-confirmed (primary; 4 studies)	0.720 (0.647–0.783)	0.883 (0.796–0.936)	19.4 (13.6–27.7)	6.16 (3.81–10.2)	0.318 (0.27–0.378)	—
Single-arm, all 5 studies (sensitivity analysis)	0.716 (0.654–0.771)	0.891 (0.820–0.937)	20.7 (14.8–29.1)	6.61 (4.24–10.5)	0.319 (0.276–0.371)	—
Single-arm, PH detection (general) stratum (2 studies)	0.732 (0.603–0.831)	0.869 (0.686–0.953)	—	—	—	—
Double-arm, Traditional (paired model; 4 studies)	0.707 (0.421–0.889)	0.640 (0.376–0.840)	4.27 (0.86–21.6)	1.93 (0.932–4.52)	0.471 (0.166–1.08)	0.019/0.0076
Double-arm, ML/DL (paired model; 4 studies)	0.838 (0.668–0.930)	0.849 (0.512–0.968)	29.3 (4.16–201)	5.54 (1.66–26.4)	0.20 (0.0824–0.453)	0.019/0.0076

CI, confidence interval; DOR, diagnostic odds ratio; PLR, positive likelihood ratio; NLR, negative likelihood ratio; RHC, right heart catheterization; PH, pulmonary hypertension; ML/DL, machine learning/deep learning; sens/spec, sensitivity/specificity. Single-arm strata are non-comparative; no between-arm test applies. Paired Wald *p* values compare ML/DL with traditional methods and are repeated across the two comparative rows for clarity. Dashes indicate metrics not reported or tests not applicable.

## Data Availability

This is a systematic review and meta-analysis of published data.

## Supplementary Materials

The following supporting information can be downloaded at https://www.mdpi.com/article/10.3390/diagnostics16193106/s1. Table S1: PRISMA-DTA checklist Item; Supplemental S2: Research question, PIRD framework, and eligibility criteria; Table S2: Search strategies; Table S3: GRADE certainty-of-evidence assessment; Table S4: Model convergence, logit-scale estimates, and continuity corrections; Table S5: Study-level diagnostic information reported in the included studies; Table S6: Full-text excluded studies and reasons for exclusion; Table S7: Per-study 2 × 2 counts and diagnostic accuracy for double-arm (comparative) studies, stratified by intended clinical task; Table S8: Comparing previous literature with current; Figure S1: QUADAS-2 risk-of-bias assessment of the included studies; Figure S2: Sensitivity analysis including all five non-comparative studies irrespective of reference standard; Figure S3: Sensitivity analysis including all five non-comparative studies irrespective of reference standard; Figure S4: Diagnostic odds ratios in the sensitivity analysis including all five non-comparative studies irrespective of reference standard; Figure S5: Positive likelihood ratios in the sensitivity analysis including all five non-comparative studies irrespective of reference standard; Figure S6: Negative likelihood ratios in the sensitivity analysis including all five non-comparative studies irrespective of reference standard; Figure S7: Pooled sensitivity within the general pulmonary hypertension detection stratum comprising Liu 2024 [13] and Salehi 2025 [14]; Figure S8: Pooled specificity within the general pulmonary hypertension detection stratum comprising Liu 2024 [13] and Salehi 2025 [14]; Figure S9: Diagnostic odds ratios for machine learning/deep learning (ML/DL) models and traditional methods in the paired comparative model; Figure S10: Positive likelihood ratios for machine learning/deep learning (ML/DL) models and traditional methods in the paired comparative model; Figure S11: Negative likelihood ratios for machine learning/deep learning (ML/DL) models and traditional methods in the paired comparative model; Figure S12: Leave-one-out analysis of the diagnostic odds ratio in the five-study non-comparative synthesis. Each row identifies the omitted study. Reference [23] is cited in the Supplementary Materials.
