# Supplementary material for: Diagnostic Accuracy of Machine Learning-Enhanced Methods for Pulmonary Hypertension: A Systematic Review and Meta-Analysis
Source: Diagnostics (Basel). 2026 Sep 24;16(19):3106. doi: 10.3390/diagnostics16193106 (PMC13664536; doi:10.3390/diagnostics16193106)
Supplement: Supplementary file 1 [file diagnostics-16-03106-s001.zip › diagnostics-4478556-supplementary.pdf]

SUPPLEMENTARY FILE

Supplementary Table S1: PRISMA-DTA checklist Item

Section/topic	#	PRISMA-DTA Checklist Item	Reported on page #
**TITLE / ABSTRACT**			
Title	1	Identify the report as a systematic review (+/- meta-analysis) of diagnostic test accuracy (DTA) studies.	Page 1
Abstract	2	Abstract: See PRISMA-DTA for abstracts.	Page 1-2
**INTRODUCTION**			
Rationale	3	Describe the rationale for the review in the context of what is already known.	Page 2-3(Introduction)
Clinical role of index test	D1	State the scientific and clinical background, including the intended use and clinical role of the index test, and if applicable, the rationale for minimally acceptable test accuracy (or minimum difference in accuracy for comparative design).	Page 2 – 3 (Introduction)
Objectives	4	Provide an explicit statement of question(s) being addressed in terms of participants, index test(s), and target condition(s).	Page 3 (final paragraph of Introduction
**METHODS**			
Protocol and registration	5	Indicate if a review protocol exists, if and where it can be accessed (e.g., Web address), and, if available, provide registration information including registration number.	Page 3 (Methods First Para)
Eligibility criteria	6	Specify study characteristics (participants, setting, index test(s), reference standard(s), target condition(s), and study design) and report characteristics (e.g., years considered, language, publication status) used as criteria for eligibility, giving rationale.	Page 3-4 (Methods; 2.2 Eligibility criteria) AND Suppl. File1.3 PIRD and 1.4.1 – 1.4.2 (page 2 – 3)
Information sources	7	Describe all information sources (e.g., databases with dates of coverage, contact with study authors to identify additional studies) in the search and date last searched.	Page 3 (Methods; 2.1. Search strategy)
Search	8	Present full search strategies for all electronic databases and other sources searched, including any limits used, such that they could be repeated.	Suppl.File Table 1.2
Study selection	9	State the process for selecting studies (i.e., screening, eligibility, included in systematic review, and, if applicable, included in the meta-analysis).	Page 3-4 (Methods; 2.2 Eligibility criteria) AND Results (3.1. Study selection)
Data collection process	10	Describe method of data extraction from reports (e.g., piloted forms, independently, in duplicate) and any processes for obtaining and confirming data from investigators.	Page 4, Methods (2.3. Data extraction)

Definitions for data extraction	11	Provide definitions used in data extraction and classifications of target condition(s), index test(s), reference standard(s) and other characteristics (e.g. study design, clinical setting).	Page 4 AND Suppl. 1.3 PIRD and 1.4.1 – 1.4.2
Risk of bias and applicability	12	Describe methods used for assessing risk of bias in individual studies and concerns regarding the applicability to the review question.	Page 4; Methods (2.4. Risk of bias assessment) AND Results (3.3. Risk of bias) AND Suppl. S2
Diagnostic accuracy measures	13	State the principal diagnostic accuracy measure(s) reported (e.g. sensitivity, specificity) and state the unit of assessment (e.g. per-patient, per-lesion).	Page 4 – 5, Methods (2.5 Statistical analysis)
Synthesis of results	14	Describe methods of handling data, combining results of studies and describing variability between studies. This could include, but is not limited to: a) handling of multiple definitions of target condition. b) handling of multiple thresholds of test positivity, c) handling multiple index test readers, d) handling of indeterminate test results, e) grouping and comparing tests, f) handling of different reference standards	Page 4 – 5, Methods (2.5 Statistical analysis); AND Page 12 – 13 Results (3.10 – 3.11)

Section/topic	#	PRISMA-DTA Checklist Item	Reported on page #
Meta-analysis	D2	Report the statistical methods used for meta-analyses, if performed.	Page 4 – 5, Methods (2.5 Statistical analysis)
Additional analyses	16	Describe methods of additional analyses (e.g., sensitivity or subgroup analyses, meta-regression), if done, indicating which were pre-specified.	Page 11, Results (3.10 and 3.11)
**RESULTS**			
Study selection	17	Provide numbers of studies screened, assessed for eligibility, included in the review (and included in meta-analysis, if applicable) with reasons for exclusions at each stage, ideally with a flow diagram.	Page 4-5; Results (3.1 Study selection) AND Figure 1
Study characteristics	18	For each included study provide citations and present key characteristics including: a) participant characteristics (presentation, prior testing), b) clinical setting, c) study design, d) target condition definition, e) index test, f) reference	Pgae 6-7; Results (3.2 Study

		standard, g) sample size, h) funding sources	characteristics) AND Table 1
Risk of bias and applicability	19	Present evaluation of risk of bias and concerns regarding applicability for each study.	Page 7; Results (3.3. Risk of bias) AND Suppl. S2
Results of individual studies	20	For each analysis in each study (e.g. unique combination of index test, reference standard, and positivity threshold) report 2x2 data (TP, FP, FN, TN) with estimates of diagnostic accuracy and confidence intervals, ideally with a forest or receiver operator characteristic (ROC) plot.	Page 8 – 10, Figures 2 – 6; Suppl Figures S3 – S5
Synthesis of results	21	Describe test accuracy, including variability; if meta-analysis was done, include results and confidence intervals.	Page 8 – 12, Results 3.3 – 3.7; Table 2 and Figures 2 – 7; Suppl. Figures S3 – S5
Additional analysis	23	Give results of additional analyses, if done (e.g., sensitivity or subgroup analyses, meta-regression; analysis of index test: failure rates, proportion of inconclusive results, adverse events).	Page 12 – 13, Results 3.8 – 3.11; Suppl. S6 GRADE tables
**DISCUSSION**			
Summary of evidence	24	Summarize the main findings including the strength of evidence.	Page 11-12, Discussion (Para 1, 4.1 and 4.2)
Limitations	25	Discuss limitations from included studies (e.g. risk of bias and concerns regarding applicability) and from the review process (e.g. incomplete retrieval of identified research).	Page 12-13, Discussion (5. Limitations)
Conclusions	26	Provide a general interpretation of the results in the context of other evidence. Discuss implications for future research and clinical practice (e.g. the intended use and clinical role of the index test).	Page 13; 6. Conclusion
**FUNDING**			
Funding	27	For the systematic review, describe the sources of funding and other support and the role of the funders.	Page 13

Adapted From: McInnes MDF, Moher D, Thombs BD, McGrath TA, Bossuyt PM, The PRISMA-DTA Group (2018). Preferred Reporting Items for a Systematic Review and Meta-analysis of Diagnostic Test Accuracy Studies: The PRISMA-DTA Statement. JAMA. 2018 Jan 23;319(4):388-396. doi: 10.1001/jama.2017.19163.

2. Supplemental S2

S2.1: RESEARCH QUESTION:

What is the diagnostic accuracy of machine learning and deep learning–based methods for detecting pulmonary hypertension compared with conventional diagnostic methods?

Supplementary Table S2: SEARCH STRATEGY:

Databases	Search Strategies	Results
PubMed	("Hypertension, Pulmonary"[Mesh] OR "pulmonary hypertension"[tiab] OR "pulmonary arterial hypertension"[tiab] OR PAH[tiab] OR "chronic thromboembolic pulmonary hypertension"[tiab] OR CTEPH[tiab] OR "persistent pulmonary hypertension of the newborn"[tiab] OR PPHN[tiab] OR "pulmonary hypertension due to left heart disease"[tiab] OR "PH-LHD"[tiab]) AND ("Artificial Intelligence"[Mesh] OR "Machine Learning"[Mesh] OR "Deep Learning"[Mesh] OR "Neural Networks, Computer"[Mesh] OR "Diagnosis, Computer-Assisted"[Mesh] OR "artificial intelligence"[tiab] OR "machine learning"[tiab] OR "deep learning"[tiab] OR "neural network*"[tiab] OR "artificial neural network*"[tiab] OR "convolutional neural network*"[tiab] OR CNN[tiab] OR "random forest*"[tiab] OR "support vector machine*"[tiab] OR "gradient boosting"[tiab] OR XGBoost[tiab] OR radiomic*[tiab] OR ResNet*[tiab] OR DenseNet*[tiab] OR transformer*[tiab] OR "computer-aided diagnos*"[tiab] OR "computer-assisted diagnos*"[tiab])	860
Scopus	TITLE-ABS-KEY(("pulmonary hypertension" OR "pulmonary arterial hypertension" OR PAH OR "chronic thromboembolic pulmonary hypertension" OR CTEPH OR "persistent pulmonary hypertension of the newborn" OR PPHN OR "pulmonary hypertension due to left heart disease" OR "PH-LHD")) AND<br><br>TITLE-ABS-KEY ("artificial intelligence" OR "machine learning" OR "deep learning" OR "neural network*" OR "artificial neural network*" OR "convolutional neural network*" OR CNN OR "random forest*" OR "support vector machine*" OR "gradient boosting" OR XGBoost OR radiomic* OR ResNet* OR DenseNet* OR transformer* OR "computer-aided diagnos*" OR "computer-assisted diagnos*")) AND<br><br>TITLE-ABS-KEY (diagnos* OR detect* OR screen* OR classif* OR discriminat* OR identif* OR predict* OR "diagnostic accuracy" OR sensitiv* OR specificit* OR "receiver operating characteristic" OR ROC OR "area under the curve" OR AUC))	1418
Embase	('pulmonary hypertension'/exp OR 'pulmonary arterial hypertension':ti,ab OR cteph:ti,ab) AND ('artificial intelligence'/exp OR 'machine learning':ti,ab OR	840

	'deep learning':ti,ab) AND ('diagnosis'/exp OR diagnos*:ti,ab OR 'diagnostic accuracy':ti,ab)	
Cochrane	#1 MeSH descriptor: [Hypertension, Pulmonary] explode all trees<br><br>#2 ("pulmonary hypertension" OR "pulmonary arterial hypertension" OR PAH OR "chronic thromboembolic pulmonary hypertension" OR CTEPH OR "persistent pulmonary hypertension of the newborn" OR PPHN OR "pulmonary hypertension due to left heart disease" OR "PH-LHD"):ti,ab,kw<br><br>#3 #1 OR #2<br><br>#4 MeSH descriptor: [Artificial Intelligence] explode all trees<br><br>#5 (Machine Learning):ti,ab,kw<br><br>#6 MeSH descriptor: [Machine Learning] explode all trees<br><br>#7 MeSH descriptor: [Neural Networks, Computer] explode all trees<br><br>#8 MeSH descriptor: [Diagnosis, Computer-Assisted] explode all trees<br><br>#9 ("artificial intelligence" OR "machine learning" OR "deep learning" OR "neural network" OR "artificial neural network" OR "convolutional neural network" OR CNN OR "random forest" OR "support vector machine" OR "gradient boosting" OR XGBoost OR radiomic OR ResNet OR DenseNet OR transformer OR "computer aided diagnos*" OR "computer assisted diagnos*"):ti,ab,kw<br><br>#10 #4 OR #5 OR #6 OR #7 OR #8 OR #9<br><br>#11 #3 AND #10	24
ScienceDirect	tak(("pulmonary hypertension" OR "pulmonary arterial hypertension") AND ("artificial intelligence" OR "machine learning" OR "deep learning") AND (diagnosis OR detection) AND ("diagnostic accuracy" OR AUC))	61

S2.3: PIRD

P (Population): Patients of any age undergoing evaluation for suspected or confirmed pulmonary hypertension, including defined PH subtypes such as pulmonary arterial hypertension and chronic thromboembolic pulmonary hypertension.
I (Index Test): Machine-learning, deep-learning, or artificial-intelligence models applied to echocardiographic, electrocardiographic, radiographic, computed-tomographic, clinical, physiological, or multimodal data for the detection, screening, or diagnosis of PH.
R (Reference Standard): Right heart catheterization using accepted study-specific hemodynamic criteria or, where RHC was impractical or not routinely performed, validated echocardiographic criteria.
D (Diagnosis of Interest): Pulmonary hypertension or a defined PH subtype.

S2.4.1: INCLUSION CRITERIA

1. Patients of any age with suspected or confirmed pulmonary hypertension (PH), including neonates and geriatric patients.
2. Studies evaluating individuals undergoing assessment for PH diagnosis or screening were eligible.
3. Studies evaluating machine learning (ML), deep learning (DL), or artificial intelligence (AI)-based algorithms for the diagnosis, screening, or detection of PH.
4. Compared with conventional diagnostic methods (e.g., clinician interpretation, standard echocardiography, conventional imaging, risk scores, or other non-AI approaches), were eligible.
5. reported at least one of the desired predetermined outcomes.
6. Prospective or retrospective cohort studies, cross-sectional diagnostic accuracy studies, and comparative diagnostic studies.

S2.4.2: EXCLUSION CRITERIA

AI/ML/DL model as the diagnostic or screening tool not evaluated.

Case reports and case series.

Reviews, editorials, letters, commentaries, and conference abstracts without sufficient data

Studies that did not report any clinically relevant diagnostic performance or outcome measures.

Studies not reporting sufficient data to extract a 2×2 contingency table or equivalent diagnostic accuracy metrics.

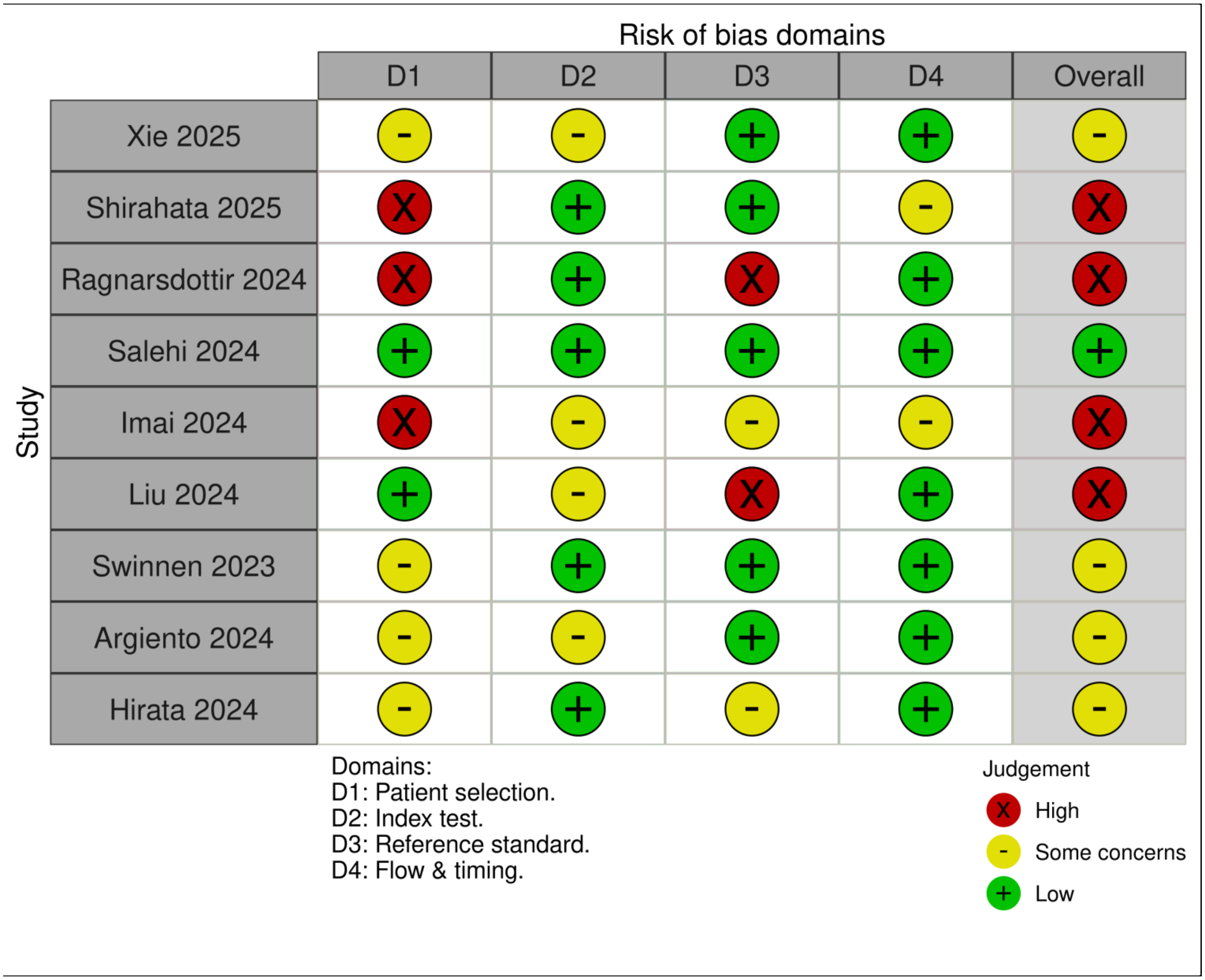

Figure S1. QUADAS-2 risk-of-bias assessment of the included studies. D1, patient selection; D2, index test; D3, reference standard; D4, flow and timing. Green, yellow and red symbols indicate judgments labelled low risk, some concerns and high risk, respectively.

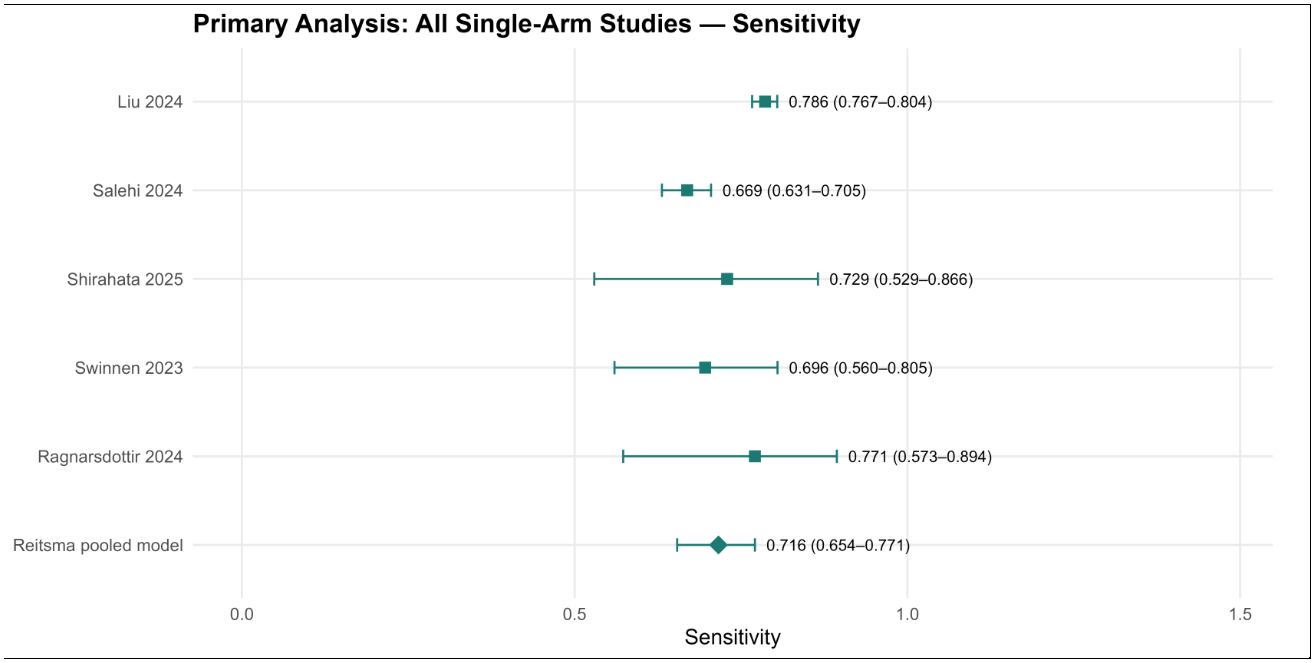

Figure S2. Sensitivity analysis including all five non-comparative studies irrespective of reference standard. Pooled sensitivity was 0.716 (95% CI: 0.654–0.771).

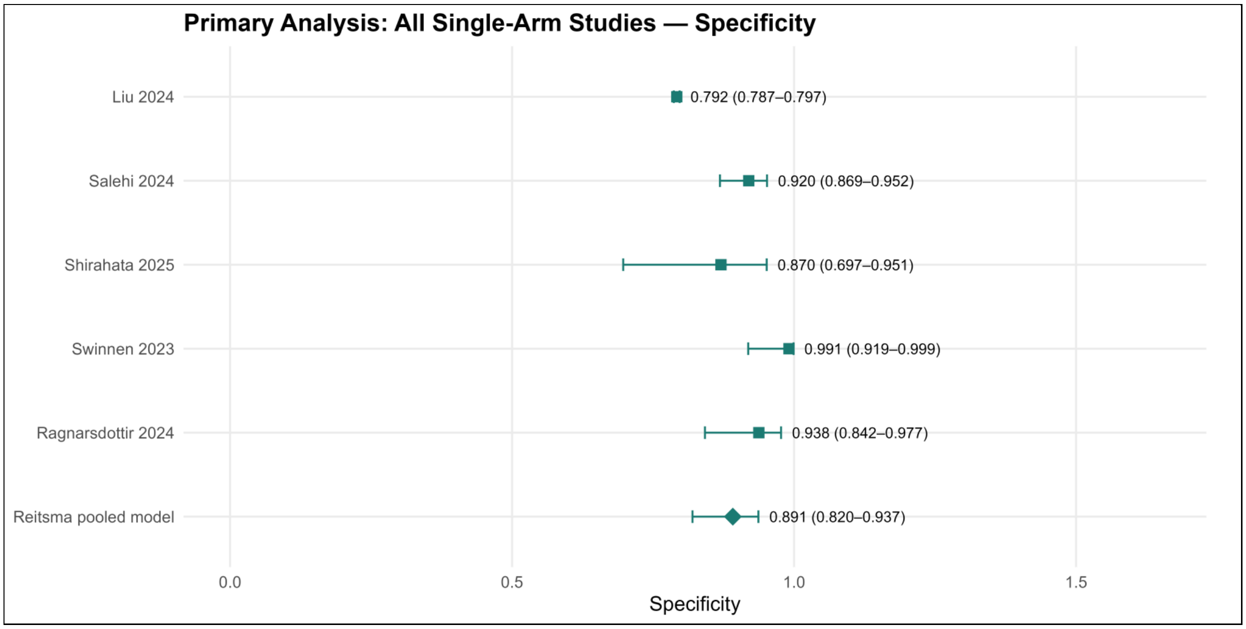

Figure S3. Sensitivity analysis including all five non-comparative studies irrespective of reference standard. Pooled specificity was 0.891 (95% CI: 0.820–0.937).

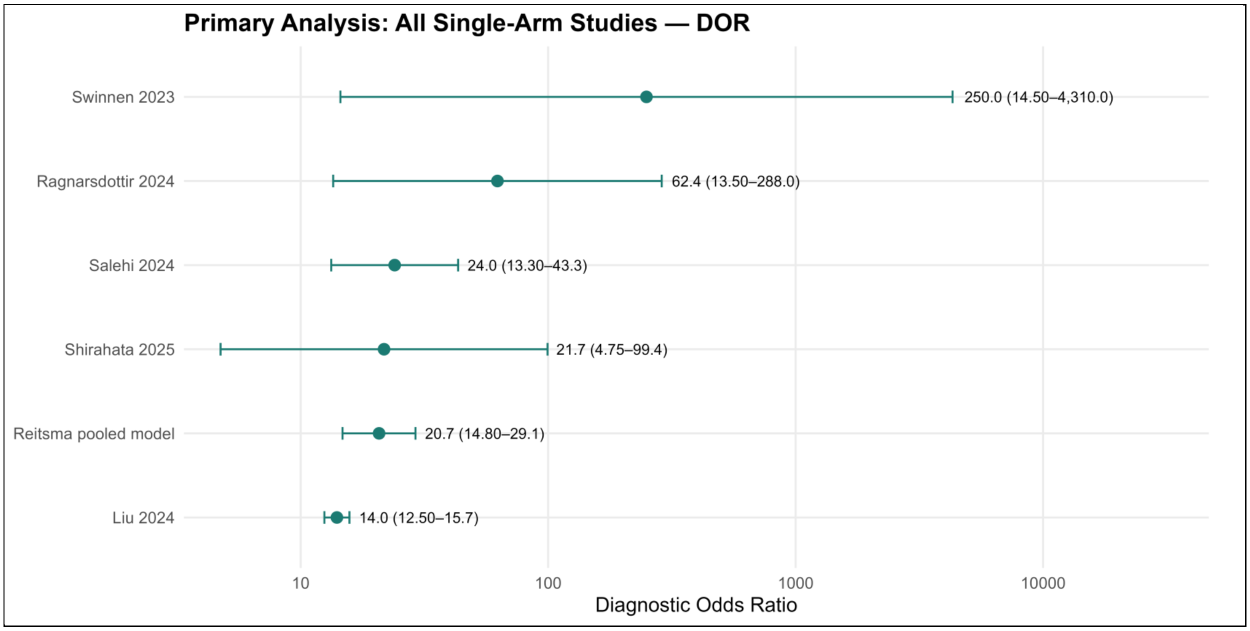

Figure S4. Diagnostic odds ratios in the sensitivity analysis including all five non-comparative studies irrespective of reference standard. The Reitsma pooled estimate was 20.7 (95% CI: 14.8–29.1). Points represent estimates and horizontal lines indicate 95% confidence intervals on a logarithmic scale.

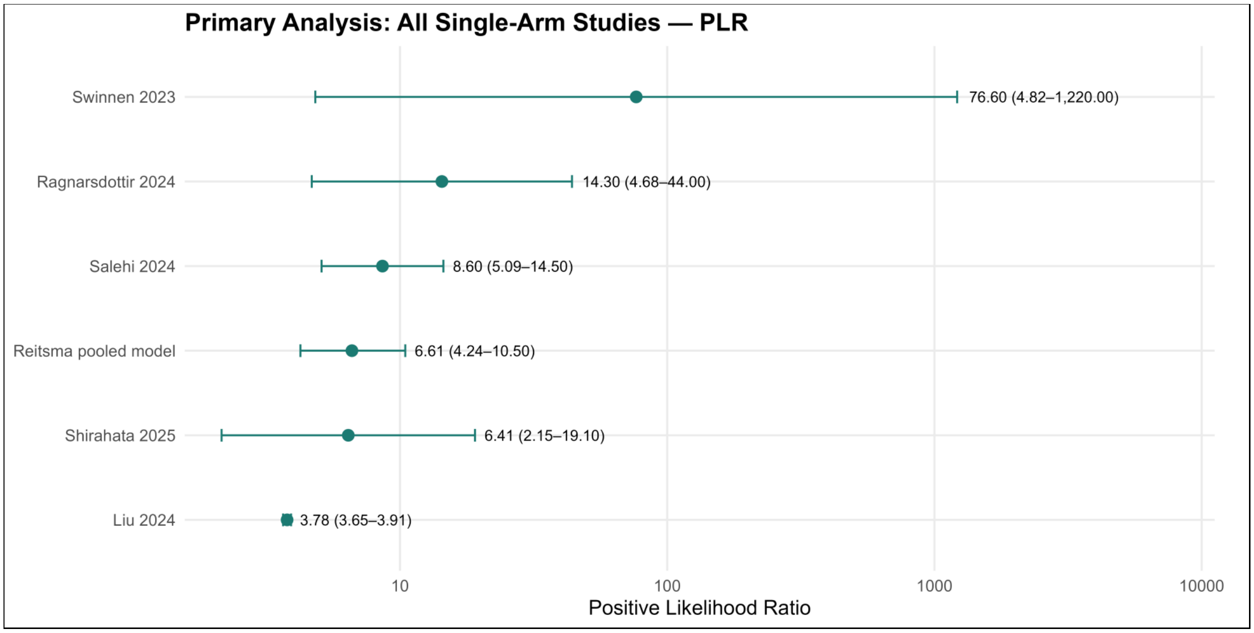

Figure S5. Positive likelihood ratios in the sensitivity analysis including all five non-comparative studies irrespective of reference standard. The Reitsma pooled estimate was 6.61 (95% CI: 4.24–10.5). Points represent estimates and horizontal lines indicate 95% confidence intervals on a logarithmic scale.

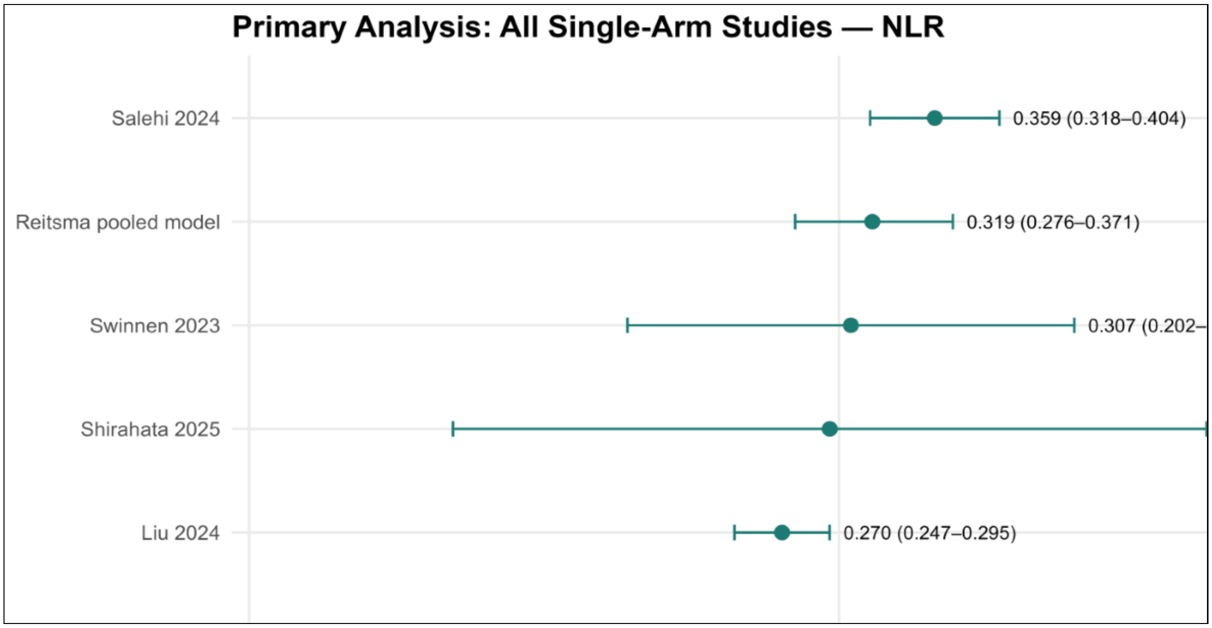

Figure S6. Negative likelihood ratios in the sensitivity analysis including all five non-comparative studies irrespective of reference standard. The Reitsma pooled estimate was 0.319 (95% CI: 0.276–0.371). Points represent estimates and horizontal lines indicate 95% confidence intervals on a logarithmic scale.

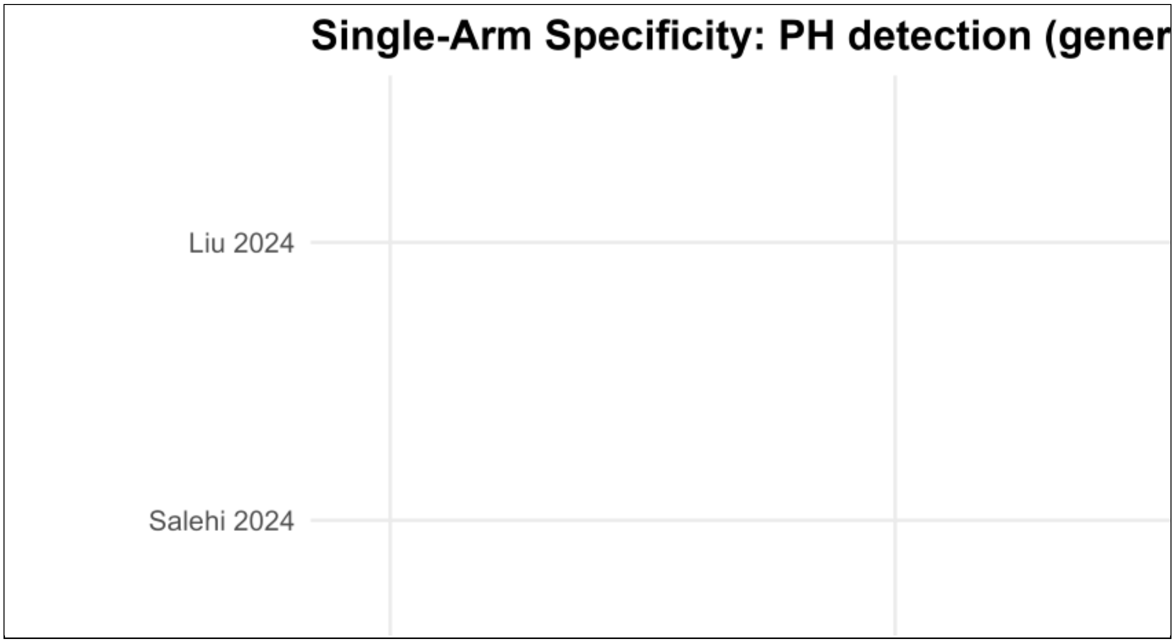

Figure S7. Pooled sensitivity within the general pulmonary hypertension detection stratum comprising Liu 2024 and Salehi 2024. The Reitsma pooled estimate was 0.732 (95% CI: 0.603–0.831). Squares represent study-level estimates, the diamond represents the pooled estimate, and horizontal lines indicate 95% confidence intervals.

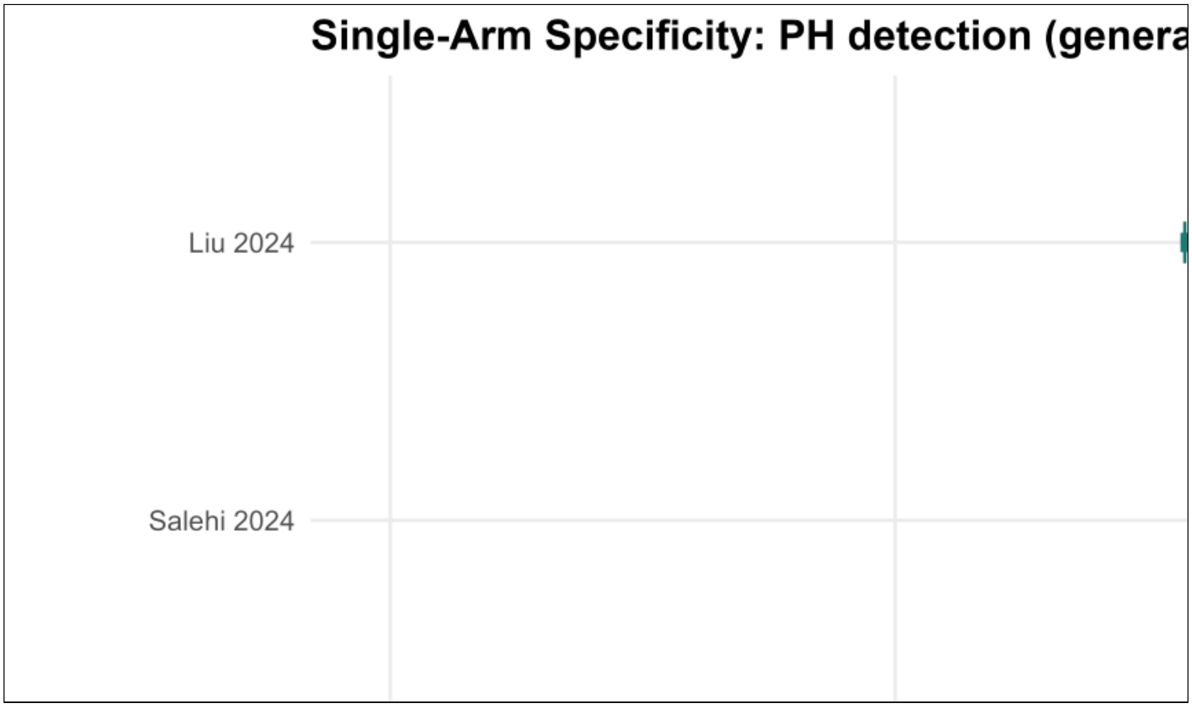

Figure S8. Pooled specificity within the general pulmonary hypertension detection stratum comprising Liu 2024 and Salehi 2024. The Reitsma pooled estimate was 0.869 (95% CI: 0.686–0.953). Squares represent study-level estimates, the diamond represents the pooled estimate, and horizontal lines indicate 95% confidence intervals.

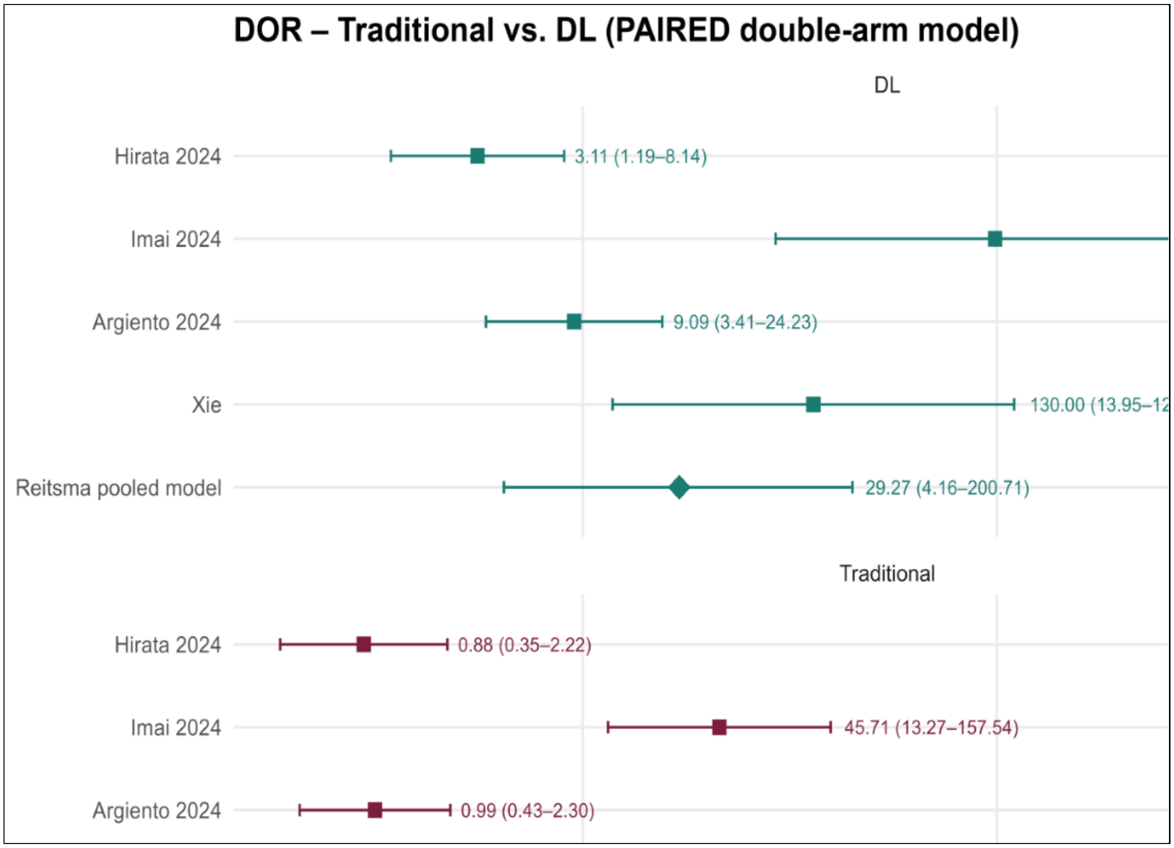

Figure S9. Diagnostic odds ratios for machine learning/deep learning (ML/DL) models and traditional methods in the paired comparative model. Pooled estimates were 29.3 (95% CI: 4.16–201) for ML/DL and 4.27 (95% CI: 0.86–21.6) for traditional methods. Teal and burgundy squares represent study-level ML/DL and traditional-method estimates, respectively; diamonds represent pooled estimates. Horizontal lines indicate 95% confidence intervals on a logarithmic scale.

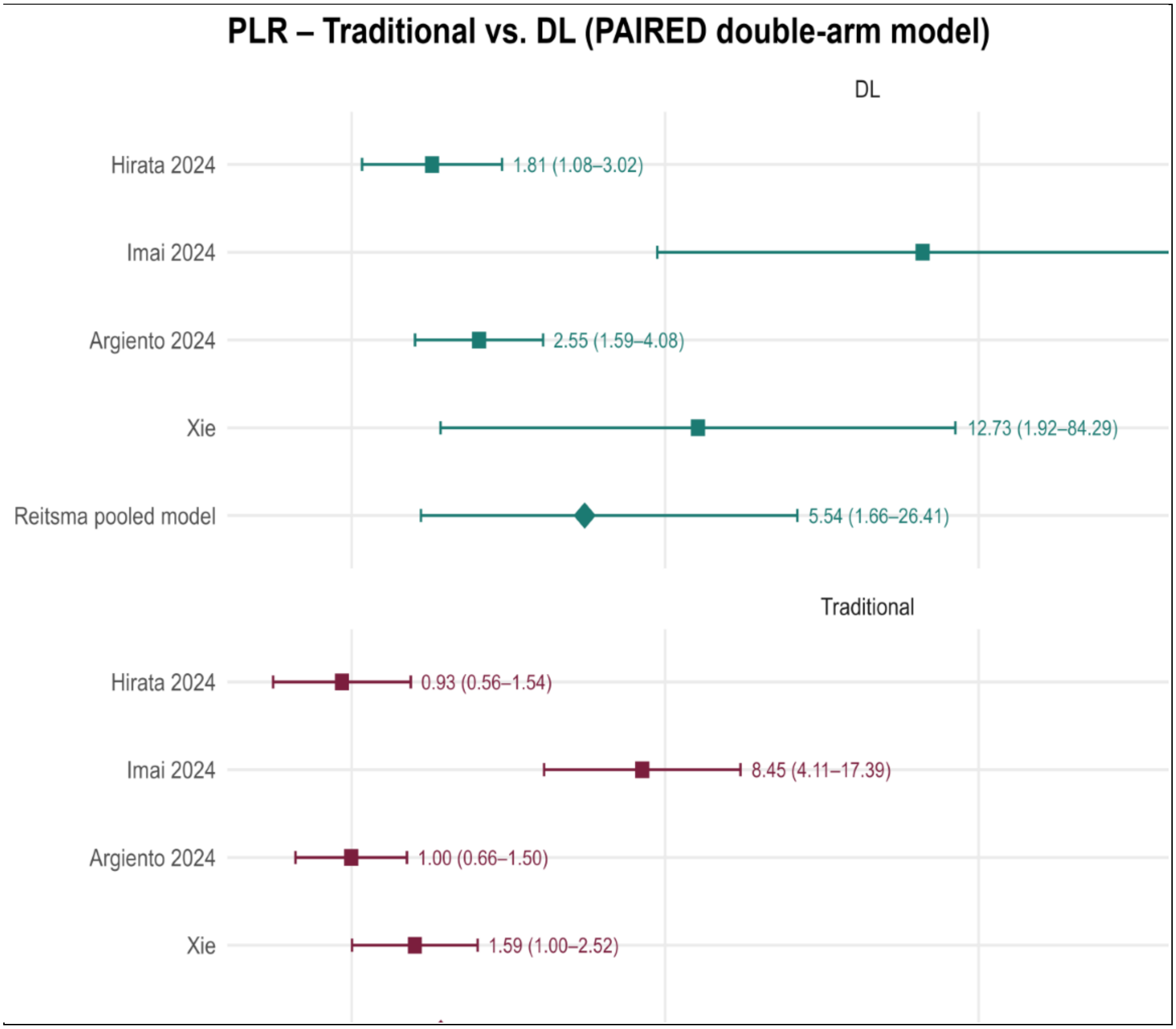

Figure S10. Positive likelihood ratios for machine learning/deep learning (ML/DL) models and traditional methods in the paired comparative model. Pooled estimates were 5.54 (95% CI: 1.66–26.4) for ML/DL and 1.93 (95% CI: 0.932–4.52) for traditional methods. Teal and burgundy squares represent study-level ML/DL and traditional-method estimates, respectively; diamonds represent pooled estimates. Horizontal lines indicate 95% confidence intervals on a logarithmic scale.

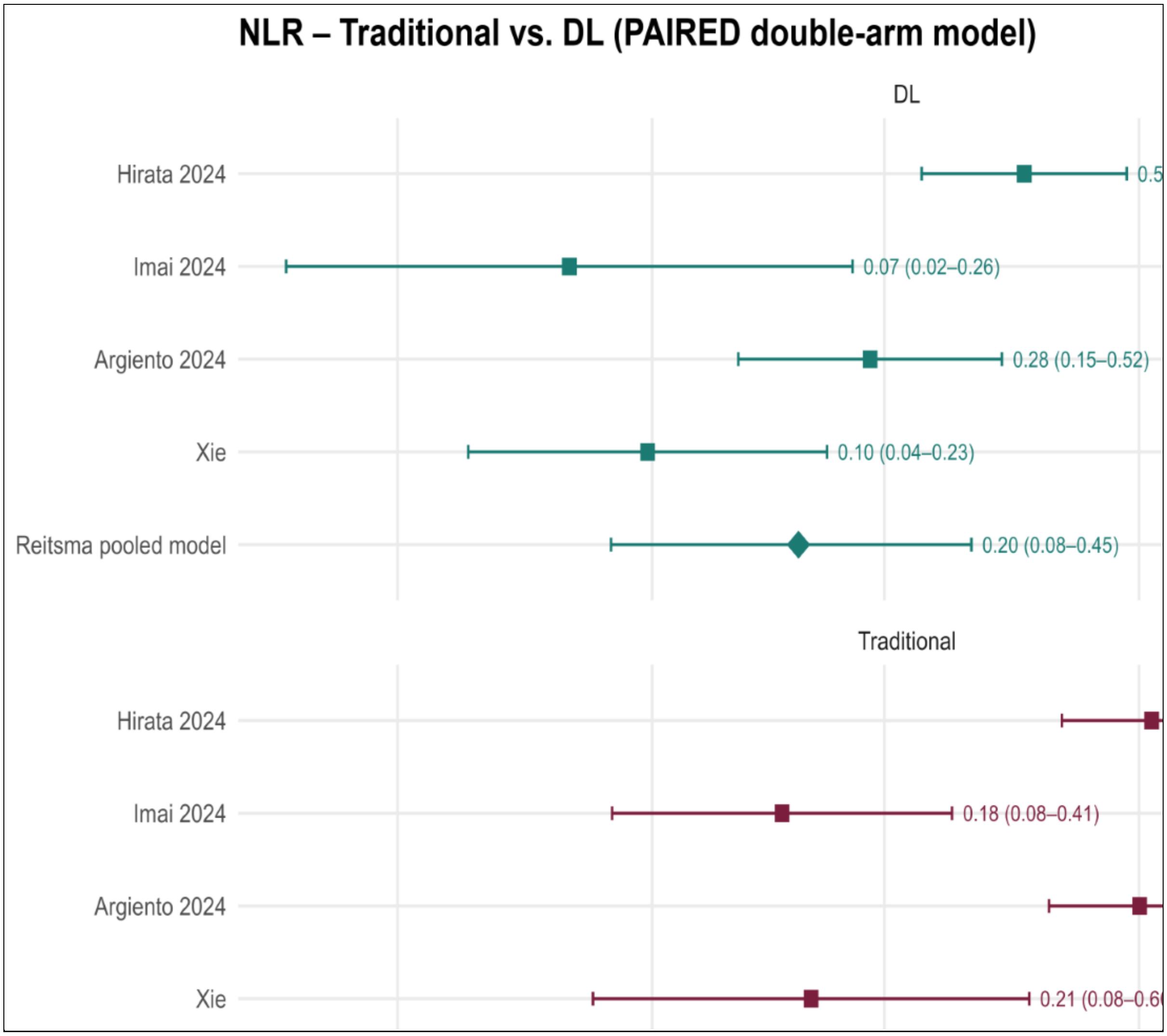

Figure S11. Negative likelihood ratios for machine learning/deep learning (ML/DL) models and traditional methods in the paired comparative model. Pooled estimates were 0.20 (95% CI: 0.0824–0.453) for ML/DL and 0.471 (95% CI: 0.166–1.08) for traditional methods. Teal and burgundy squares represent study-level ML/DL and traditional-method estimates, respectively; diamonds represent pooled estimates. Horizontal lines indicate 95% confidence intervals on a logarithmic scale.

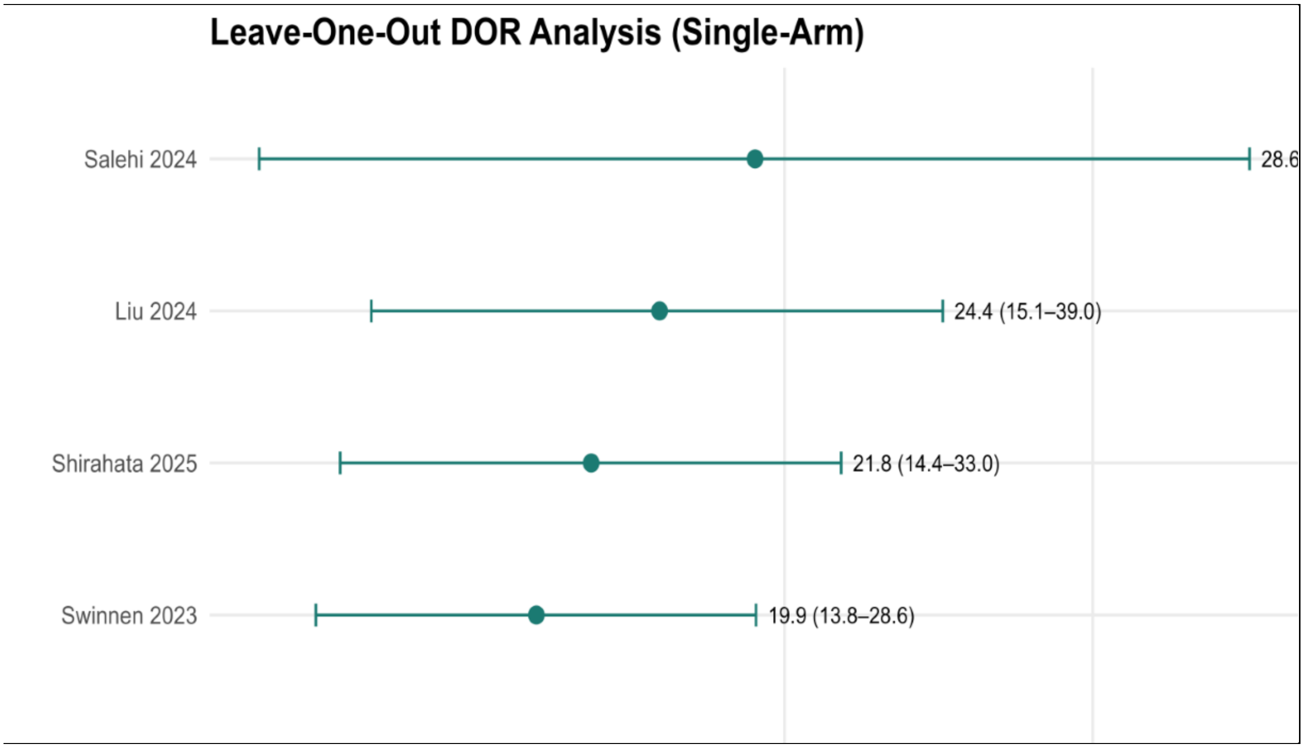

Figure S12. Leave-one-out analysis of the diagnostic odds ratio in the five-study non-comparative synthesis. Each row identifies the omitted study; points and horizontal lines show the recalculated pooled estimate and its 95% confidence interval. Point estimates ranged from 19.4 after excluding Ragnarsdóttir 2024 to 28.6 after excluding Salehi 2024, within the full-model confidence interval of 14.8–29.1.

7. Supplemental Table S3: GRADE certainty-of-evidence assessment

GRADE Certainty of Evidence Assessment

Outcome	No. of Participants (Studies)	Risk of Bias	Inconsistency	Indirectness	Imprecision	Publication Bias	Certainty of Evidence (GRADE)
**Single-arm (RHC-confirmed, primary)**							
Sensitivity	26,452(4)	Serious[a]	Serious[b]	Not serious[c]	Not Serious	Undetected[d]	⊕⊕◯◯ (Low)
Specificity	26,452(4)	Serious[a]	Serious[b]	Not serious[c]	Not Serious	Undetected[d]	⊕⊕◯◯ (Low)
**Double-arm – Traditional (paired model)**							
Sensitivity	330(4)	Serious[a]	Serious[b]	Not serious[c]	Serious[e]	Undetected[d]	⊕◯◯◯ (Very low)
Specificity	330(4)	Serious[a]	Serious[b]	Not serious[c]	Serious[e]	Undetected[d]	⊕◯◯◯ (Very low)
**Double-arm – DL (paired model)**							
Sensitivity	330(4)	Serious[a]	Serious[b]	Not serious[c]	Not Serious	Undetected[d]	⊕⊕◯◯ (Low)
Specificity	330(4)	Serious[a]	Serious[b]	Not serious[c]	Serious[e]	Undetected[d]	⊕◯◯◯ (Very low)

a Due to high risk of bias identified in one or more included studies (QUADAS-2)

b High heterogeneity I^2 >50%

c Population, index test, and reference standard consistent across studies

[d] Number of studies insufficient to formally assess publication bias

[e] Due to wide confidence intervals

Supplemental Table S4: Model convergence, logit-scale estimates, and continuity corrections

A) Single-arm Reitsma bivariate models

Model / analysis	Converged	Logit sensitivity (SE)	Logit specificity (SE)	Correlation (sens–spec)	Zero-cell correction
**Single-arm: PH detection (general)**	Yes	1.006 (0.300)	1.893 (0.568)	0.332	None
**RHC-confirmed primary analysis**	Yes	0.945 (0.173)	2.019 (0.335)	0.173	Swinnen 2023 (0.5-cell)
**All single-arm studies (primary, n=5)**	Yes	0.925 (0.147)	2.105 (0.301)	0.152	Swinnen 2023 (0.5-cell)
**LOO excluding Liu 2024**	Yes	0.735 (0.080)	2.457 (0.228)	<0.001	Swinnen 2023 (0.5-cell)
**LOO excluding Salehi 2024**	Yes	1.106 (0.121)	2.243 (0.518)	0.163	Swinnen 2023 (0.5-cell)
**LOO excluding Shirahata 2025**	Yes	0.903 (0.174)	2.176 (0.368)	0.195	Swinnen 2023 (0.5-cell)
**LOO excluding Swinnen 2023**	Yes	0.958 (0.168)	2.031 (0.336)	0.168	None (Swinnen excluded)

**LOO excluding Ragnarsdóttir 2024***	Yes	0.945 (0.173)	2.019 (0.335)	0.173	Swinnen 2023 (0.5-cell)
**External-validation-only subset**	Yes	1.103 (0.146)	2.576 (0.860)	0.287	Swinnen 2023 (0.5-cell)
**Internal-validation-only subset**	Yes	0.716 (0.084)	2.393 (0.261)	<0.001	None

B) Paired double-arm bivariate models (traditional vs. ML/DL arms)

Model / analysis	Converged (sens/spec)	Traditional logit-sens (SE)	ML/DL logit-sens (SE)	Traditional logit-spec (SE)	ML/DL logit-spec (SE)	Correction
**All double-arm studies (primary paired model)**	Yes / Yes	0.879 (0.612)	1.640 (0.481)	0.577 (0.553)	1.731 (0.858)	10^{-4} clamp
**Age band: younger (≤ median)**	Yes / Yes	1.969 (0.367)	2.412 (0.402)	0.987 (1.254)	3.408 (1.088)	10^{-4} clamp
**Age band: older (> median)**	Yes / Yes	−0.091 (0.253)	0.954 (0.487)	0.025 (0.225)	0.712 (0.240)	10^{-4} clamp
**LOO excluding Hirata 2024**	Yes / Yes	1.285 (0.687)	2.025 (0.396)	0.726 (0.750)	2.196 (1.135)	10^{-4} clamp
**LOO excluding Imai 2024**	Yes / Yes	0.652 (0.788)	1.403 (0.549)	−0.021 (0.208)	0.805 (0.233)	10^{-4} clamp
**LOO excluding Argiento 2024**	Yes / Yes	1.213 (0.774)	1.688 (0.664)	0.757 (0.740)	2.211 (1.154)	10^{-4} clamp

**LOO excluding Xie 2025**	Yes / Yes	0.405 (0.554)	1.510 (0.668)	0.746 (0.739)	1.801 (1.130)	10^{-4} clamp

Supplementary Table S5. Study-level diagnostic information reported in the included studies

Study	Validation/test cohort	Modality / model	Population / reference standard	Threshold / rule	TP	FP	FN	TN	Reported results
**Swinnen et al. [12]**	Internal test n=104; external validation n=165 (91 PAH, 74 PH-LHD)	Echocardiography + ECG + PFT; random forest	PAH vs PH-LHD; RHC	Probability 0.5	-	-	-	-	Internal test: 35/50 PH-LHD correctly diagnosed; PPV 100%, NPV 78%, specificity 100%. External validation: sensitivity 64%, PPV 100%, NPV 78%, specificity 100%.
**Liu et al. [13]**	Internal validation n=25,512; external validation n=16,736	ECG + chest X-ray; CNN/DenseNet-121 + XGBoost	PAH/CTEPH; RHC (mPAP >20)	Youden-index cutoff; numeric cutoff not reported	-	-	-	-	Internal combined ECG+CXR: sensitivity 0.786; specificity 0.792. External combined ECG+CXR: sensitivity 0.816; specificity 0.781.

**Salehi et al. [14]**	787 with mPAP; 732 with mPAP + PVR	Echocardiography; US2.AI automated TRJV	PH; RHC	TRJV 2.8 and 3.4 m/s	-	-	-	-	Automated TRJV, PH: AUC 0.88. At 2.8 m/s: sensitivity 0.89, specificity 0.63, accuracy 0.83. At 3.4 m/s: sensitivity 0.67, specificity 0.92, accuracy 0.72.
**Shirahata et al. [15]**	n=163; 70% training / 30% test	CT; random forest using automated central CT measures	PH in idiopathic pulmonary fibrosis; RHC	-	-	-	-	-	Best PA-volume RF model: AUC 0.866, sensitivity 0.739, specificity 0.885, accuracy 0.816. RV/LV ratio RF model: AUC 0.88, sensitivity 0.83, specificity 0.73.
**Ragnarsdóttir et al. [16]**	192 training/validation + held-out test n=78	Echocardiography; 3D deep-learning CNN	Neonatal PH; echocardiographic criteria	-	-	-	-	-	Held-out test performance was reported using model-level measures including AUROC

									and F1; no study-level TP/FP/FN/TN table was reported in the shared material.
**Hirata et al. [17]**	Derivation n=720; prospective validation n=165	Echocardiography; elastic-net logistic regression	Non-PH / precapillary PH / postcapillary PH; RHC	Multiclass; no single binary cutoff	-	-	-	-	Derivation: AUC 0.789 (normal), 0.766 (precapillary PH), 0.742 (postcapillary PH). Accuracy 59.4% vs 51.6% for guideline-based assessment. Prospective validation: 59.4% vs 57.8%, p=0.638.
**Imai et al. [18]**	Test: 87 patients / 101 images	Chest X-ray; ResNet50 deep learning	PAH; RHC	AUC-derived threshold 0.69	-	-	-	-	Sensitivity and specificity were reported for the test cohort; no directly reported TP/FP/FN/TN table was identified in the shared study material.

**Argiento et al. [19]**	Training n=138; independent test n=88	Echocardiography + clinical history; regularized regression	PH; RHC	Risk-score cutoff 2.155	-	-	-	-	Independent test: AUC 83% (95% CI 63–84%), sensitivity 65–94% (95% CI), specificity 53–83% (95% CI), overall accuracy 74% (95% CI 61–84%).
**Xie et al. [20]**	n=345; 80% training / 20% test; 271 PH and 74 without PH overall	CT pulmonary angiography; radiomic-feature XGBoost model	PH; RHC	Optimal operating threshold shown in confusion matrix	53	5	5	10	Radiomics model confusion matrix: TP 53, FP 5, FN 5, TN 10. AUC-PR 0.99; sensitivity 91%; specificity 91%. PAWP prediction ICC 0.87; mPAP ICC 0.74; PVR ICC 0.73.

Abbreviations: AUC, area under the curve; AUC-PR, area under the precision–recall curve; CI, confidence interval; CXR, chest X-ray; ECG, electrocardiography; FN, false negative; FP, false positive; PFT, pulmonary function testing; PH, pulmonary hypertension; PH-LHD, pulmonary hypertension due to left heart disease; RHC, right heart catheterization; TN, true negative; TP, true positive; TRJV, tricuspid regurgitation jet velocity.

Supplementary Table S6: Full-Text Excluded Studies

A) Reason of exclusion of each study.

So.no	Title	Year	Doi	Link	Reason of exclusion
1	Can Artificial Intelligence Substitute Right-Heart Catheterization With Chest X-Rays?	2021	10.1016/j.cjca.2021.03.004	https://doi.org/10.1016/j.cjca.2021.03.004	Wrong publication type (commentary)
2	Overcoming Diagnostic Delays in Pulmonary Hypertension With Deep-Learning ECG Analysis	2023	10.1016/j.cardfail.2023.02.007	https://doi.org/10.1016/j.cardfail.2023.02.007	Wrong publication type (commentary)
3	Noninvasive diagnostic modalities and prediction models for detecting pulmonary hypertension associated with interstitial lung disease	2024	10.1183/16000617.0092-2024	https://doi.org/10.1183/16000617.0092-2024	Wrong study design (narrative review)
4	Cardiac Magnetic Resonance in Pulmonary Hypertension - an Update	2020	10.1007/s12410-020-09550-2	https://doi.org/10.1007/s12410-020-09550-2	Wrong study design (narrative review)
5	Emerging multimodality imaging techniques for the pulmonary circulation	2024	10.1183/13993003.01128-2024	https://doi.org/10.1183/13993003.01128-2024	Wrong study design (narrative review)
6	Deep Learning-Based Pulmonary Artery Surface Mesh Generation	2024	10.1007/978-3-031-52448-6_14	https://doi.org/10.1007/978-3-031-52448-6_14	Wrong index test output (segmentation only, no PH classification)
7	Quantitative texture analysis using machine learning for predicting interpretable pulmonary perfusion from non-contrast computed tomography	2024	10.1186/s12931-024-03004-9	https://doi.org/10.1186/s12931-024-03004-9	Wrong population (pulmonary embolism, not PH)
8	TVnet: Automated Time-Resolved Tracking of the	2021	10.1007/978-3-030-87231-1_55	https://doi.org/10.1007/978-3-030-87231-1_55	Wrong index test output (valve-plane tracking only, no PH classification)

	Tricuspid Valve Plane in MRI Long-Axis Cine Images				
**9**	Machine learning algorithms estimating prognosis and guiding therapy in adult congenital heart disease: Data from a single tertiary centre including 10 019 patients	2019	10.1093/eurheartj/ehy915	https://doi.org/10.1093/eurheartj/ehy915	Wrong outcome (not diagnostic accuracy)
**10**	Continuous and noninvasive estimation of right ventricle systolic blood pressure using heart sound signal by deep bidirectional LSTM network	2020	10.3390/APP10165466	https://doi.org/10.3390/APP10165466	Wrong outcome (not diagnostic accuracy)
**11**	Association between the albumin–bilirubin (ALBI) score and severity of portopulmonary hypertension (PoPH): A data-mining analysis	2021	10.1111/hepr.13714	https://doi.org/10.1111/hepr.13714	Wrong outcome (not diagnostic accuracy)
**12**	Evaluation of Effect of Curcumin on Psychological State of Patients with Pulmonary Hypertension by Magnetic Resonance Image under Deep Learning	2021	10.1155/2021/9935754	https://doi.org/10.1155/2021/9935754	Wrong outcome (prognosis, not diagnostic accuracy)
**13**	AI-enabled epidermal electronic system to automatically monitor a prognostic parameter for hypertension with a smartphone	2023	10.1016/j.bios.2023.115693	https://doi.org/10.1016/j.bios.2023.115693	Wrong outcome (prognosis, not diagnostic accuracy)
**14**	Chest Computed Tomography Machine	2023	10.1164/ajrccm-conference.2023.D91	https://doi.org/10.1164/ajrccm-conference.2023.D91	Wrong outcome (severity, not diagnostic accuracy)

	Learning Classifier for Idiopathic Pulmonary Fibrosis Predicts Mortality in Interstitial Lung Diseases				
**15**	A Machine Learning Approach to Identify Patients at Risk for Long-Term Consequences after Pulmonary Embolism	2024	10.1055/s-0044-1779064	https://doi.org/10.1055/s-0044-1779064	Wrong outcome (severity, not diagnostic accuracy)
**16**	A machine learning approach to predict mortality and neonatal persistent pulmonary hypertension in newborns with congenital diaphragmatic hernia. A retrospective observational cohort study	2025	10.1007/s00431-025-06073-0	https://doi.org/10.1007/s00431-025-06073-0	Wrong outcome (severity/mortality, not diagnostic accuracy)
**17**	Applications of artificial intelligence in computed tomography imaging for phenotyping pulmonary hypertension	2024	10.1097/MCP.0000000000001103	https://doi.org/10.1097/MCP.0000000000001103	Wrong outcome (severity, not diagnostic accuracy)
**18**	CT Emphysema Subtypes and Cardiac Hemodynamics Estimated on Magnetic Resonance Imaging: The MESA COPD Study	2025	10.1016/j.chest.2025.03.009	https://doi.org/10.1016/j.chest.2025.03.009	Wrong outcome (prognosis, not diagnostic accuracy)
**19**	Elevated 15-lipoxygenase in Circulating Exosomes as a Prognostic Biomarker in Idiopathic Pulmonary Fibrosis Patients Stratified by Normal Lung Area and Tricuspid Regurgitation Velocity	2025	10.1164/ajrccm.2025.211.Abstracts.A5342	https://doi.org/10.1164/ajrccm.2025.211.Abstracts.A5342	Wrong outcome (severity, not diagnostic accuracy)

**20**	ES08.07 System Approach to Screening Management	2019	10.1016/j.jtho.2019.08.105	https://doi.org/10.1016/j.jtho.2019.08.105	Wrong publication type (conference abstract)
**21**	Value of imaging	2019	10.1002/ppul.24371	https://doi.org/10.1002/ppul.24371	Wrong publication type (proceeding)
**22**	Distal pulmonary vascular pruning in acute pulmonary embolism and chronic thromboembolic pulmonary hypertension: A comparison	2020	Not available	https://www.embase.com/search/results?subaction=viewrecord&id=L632376211&from=export	Wrong publication type (conference abstract)
**23**	Automated Segmentation of the Right Ventricle from Magnetic Resonance Imaging Using Deep Convolutional Neural Networks	2022	10.1007/978-3-030-93722-5_37	https://doi.org/10.1007/978-3-030-93722-5_37	Wrong publication type (conference abstract)
**24**	HRCT chest analysis for detection of pulmonary arterial hypertension in COVID-19 patients using convolutional neural networks	2022	10.1109/GCAT55367.2022.9972033	https://doi.org/10.1109/GCAT55367.2022.9972033	Wrong publication type (conference abstract/proceeding)
**25**	Artificial Intelligence Applied to Chest Computed Tomography: Pulmonary Artery Volume as an Image Marker of Pulmonary Hypertension	2024	Not available	https://www.embase.com/search/results?subaction=viewrecord&id=L644435642&from=export	Wrong publication type (conference abstract)
**26**	Automated CT Quantification of Changes to the Heart and Pulmonary Artery Post PTE in Patients With CTEPH	2024	Not available	https://www.embase.com/search/results?subaction=viewrecord&id=L644438848&from=export	Wrong publication type (conference abstract)
**27**	Deep Learning Quantification of Unilateral Air Trapping and Bronchiolitis Obliterans Syndrome	2024	Not available	https://www.embase.com/search/results?subaction=viewrecord&id=L644441664&from=export	Wrong publication type (proceeding)

	Post Lung Transplantation				
**28**	Diagnosis of Chronic Obstructive Pulmonary Disease Using Deeplearning on Electrocardiograms	2024	Not available	https://www.embase.com/search/results?subaction=viewrecord&id=L644439613&from=export	Wrong publication type (conference abstract)
**29**	CT Based Arterial and Venous Morphologic Changes In Pulmonary Hypertension Associated with COPD	2018	10.1183/13993003.congress-2018.PA3105	https://doi.org/10.1183/13993003.congress-2018.PA3105	Wrong reference standard
**30**	The National Echocardiography Database Australia (NEDA): Rationale and methodology	2018	10.1016/j.ahj.2018.07.001	https://doi.org/10.1016/j.ahj.2018.07.001	Wrong reference standard
**31**	A deep learning based automatic classification algorithms used for pulmonary veins and arteries separation in CT images	2019	Not available	https://www.scopus.com/inward/record.uri?eid=2-s2.0-85068464076&partnerID=40&md5=7332bb5ed77264a9f2978a604dac2149	Wrong reference standard
**32**	Automated and Interpretable Patient ECG Profiles for Disease Detection, Tracking, and Discovery	2019	10.1161/CIRCOUTCOMES.118.005289	https://doi.org/10.1161/CIRCOUTCOMES.118.005289	Wrong reference standard
**33**	Screening strategies for pulmonary arterial hypertension	2019	10.1093/eurheartj/suz204	https://doi.org/10.1093/eurheartj/suz204	Wrong reference standard
**34**	Statement on imaging and pulmonary hypertension from the Pulmonary Vascular Research Institute (PVRI)	2019	10.1177/2045894019841990	https://doi.org/10.1177/2045894019841990	Wrong reference standard
**35**	AI-based radiomic approach in high-resolution CT images for	2020	10.1007/s11548-020-02171-6	https://doi.org/10.1007/s11548-020-02171-6	Wrong reference standard

	differential diagnosis of idiopathic pulmonary fibrosis				
**36**	Machine Learning and Deep Neural Network Applications in the Thorax: Pulmonary Embolism, Chronic Thromboembolic Pulmonary Hypertension, Aorta, and Chronic Obstructive Pulmonary Disease	2020	10.1097/RTI.0000000000000492	https://doi.org/10.1097/RTI.0000000000000492	Wrong reference standard
**37**	Semi-automated Analysis of Tricuspid Regurgitation Doppler Profile for Detection and Evaluation of Pulmonary Hypertension	2020	10.1161/circ.142.suppl_3.14250	https://doi.org/10.1161/circ.142.suppl_3.14250	Wrong reference standard
**38**	An AI Based Detection System of "At Risk" Aortic Stenosis	2022	10.1016/j.hlc.2022.06.215	https://doi.org/10.1016/j.hlc.2022.06.215	Wrong reference standard
**39**	Extraction of mediastinal great vessels from noncontrast CT images using 3D U-Net and its application to CTEPH	2022	10.1007/s11548-022-02635-x	https://doi.org/10.1007/s11548-022-02635-x	Wrong reference standard
**40**	Integrated Cardiopulmonary MRI Assessment of Pulmonary Hypertension	2022	10.1002/jmri.27849	https://doi.org/10.1002/jmri.27849	Wrong reference standard
**41**	Novel Approaches to Imaging the Pulmonary Vasculature and Right Heart	2022	10.1161/CIRCRESAHA.121.319990	https://doi.org/10.1161/CIRCRESAHA.121.319990	Wrong reference standard
**42**	The Match of Electrocardiogram and Artificial Intelligence Confers an Opportunity for Pulmonary Hypertension Screening	2022	10.1016/j.jacasi.2022.03.006	https://doi.org/10.1016/j.jacasi.2022.03.006	Wrong reference standard

**43**	AI-based Clot Burden Detection and Assessment for CTEPH Severity	2023	10.1164/ajrccm-conference.2023.A26	https://doi.org/10.1164/ajrccm-conference.2023.A26	Wrong reference standard
**44**	ARTIFICIAL INTELLIGENCE ALGORITHM FOR THE DETECTION OF ATRIAL SEPTAL DEFECT USING ELECTROCARDIOGR AM	2023	10.1016/S0735-1097(23)02798-5	https://doi.org/10.1016/S0735-1097(23)02798-5	Wrong reference standard
**45**	Application of Artificial Intelligence-Based Auxiliary Diagnosis in Congenital Heart Disease Screening	2023	10.14744/AnatolJCardiol.2022.1386	https://doi.org/10.14744/AnatolJCardiol.2022.1386	Wrong reference standard
**46**	Chronic thromboembolic pulmonary hypertension: realising the potential of multimodal management	2023	10.1016/S2213-2600(23)00292-8	https://doi.org/10.1016/S2213-2600(23)00292-8	Wrong reference standard
**47**	Detection of pulmonary hypertension associated with congenital heart disease based on time-frequency domain and deep learning features	2023	10.1016/j.bspc.2022.104316	https://doi.org/10.1016/j.bspc.2022.104316	Wrong reference standard
**48**	EfDenseNet: Automated Pulmonary Hypertension Detection Model Based on EfficientNetb0 and DenseNet201 Using CT Images	2023	10.1109/ACCESS.2023.3338228	https://doi.org/10.1109/ACCESS.2023.3338228	Wrong reference standard
**49**	Evolution and optimization of clinical trial endpoints and design in pulmonary arterial hypertension	2023	10.1002/pul2.12271	https://doi.org/10.1002/pul2.12271	Wrong reference standard
**50**	Machine Learning, Pulmonary Hypertension…	2023	10.14744/AnatolJCardiol.2023.11	https://doi.org/10.14744/AnatolJCardiol.2023.11	Wrong reference standard

**51**	Next Generation ECG: The Impact of Artificial Intelligence and Machine Learning	2023	10.1007/s12170-023-00723-4	https://doi.org/10.1007/s12170-023-00723-4	Wrong reference standard
**52**	SEGMENTATION AND CLASSIFICATION OF THE PULMONARY VASCULAR SYSTEM: A GRAPH THEORY APPROACH	2023	10.1016/j.ejmp.2023.102744	https://doi.org/10.1016/j.ejmp.2023.102744	Wrong reference standard
**53**	The Echocardiographic Evaluation of the Right Heart: Current and Future Advances	2023	10.1007/s11886-023-02001-6	https://doi.org/10.1007/s11886-023-02001-6	Wrong reference standard
**54**	A Novel EMR-Based Algorithm with the Virtual Echocardiography Screening Tool (VEST) to Screen Patients for Pulmonary Arterial Hypertension	2024	10.1161/circ.150.suppl_1.4144283	https://doi.org/10.1161/circ.150.suppl_1.4144283	Wrong reference standard
**55**	Artificial intelligence meets pulmonary hypertension: early detection for a late-presenting disease	2024	10.1183/13993003.01138-2024	https://doi.org/10.1183/13993003.01138-2024	Wrong reference standard
**56**	SCALED SIAMESE CNN-BASED AUTOMATIC CLASSIFICATION ALGORITHM FOR DETECTING PULMONARY EMBOLISM	2024	10.4015/S1016237224500285	https://doi.org/10.4015/S1016237224500285	Wrong reference standard
**57**	Screening and diagnosis of cardiovascular disease using artificial intelligence-enabled cardiac magnetic resonance imaging	2024	10.1038/s41591-024-02971-2	https://doi.org/10.1038/s41591-024-02971-2	Wrong reference standard

**58**	Development and Evaluation of a Deep Learning–Based Pulmonary Hypertension Screening Algorithm Using a Digital Stethoscope	2025	10.1161/JAHA.124.036882	https://doi.org/10.1161/JAHA.124.036882	Wrong reference standard
**59**	State of precision medicine for heart failure with preserved ejection fraction in a new therapeutic age	2025	10.1002/ehf2.15205	https://doi.org/10.1002/ehf2.15205	Wrong reference standard
**60**	The Role of Computed Tomography and Artificial Intelligence in Evaluating the Comorbidities of Chronic Obstructive Pulmonary Disease: A One-Stop CT Scanning for Lung Cancer Screening	2025	10.2147/COPD.S508775	https://doi.org/10.2147/COPD.S508775	Wrong reference standard
**61**	The use of imaging in the diagnosis and treatment of thromboembolic pulmonary hypertension	2025	10.5603/cj.102716	https://doi.org/10.5603/cj.102716	Wrong reference standard
**62**	CIPHER AND CIPHER-MRI: TWO PROSPECTIVE, MULTICENTER STUDIES FOR THE IDENTIFICATION OF BIOMARKER SIGNATURES FOR EARLY DETECTION OF PULMONARY HYPERTENSION	2020	10.1016/j.chest.2020.08.1876	https://doi.org/10.1016/j.chest.2020.08.1876	Wrong index test
**63**	A diagnostic miRNA signature for pulmonary arterial hypertension using a consensus	2021	10.1016/j.ebiom.2021.103444	https://doi.org/10.1016/j.ebiom.2021.103444	Wrong index test

	machine learning approach				
**64**	Detection of the gene expressions in peripheral blood involved in the progression of pulmonary vessel disease at the subclinical stage of pulmonary hypertension associated with systemic sclerosis	2021	10.1136/annrheumdis-2021-eular.3609	https://doi.org/10.1136/annrheumdis-2021-eular.3609	Wrong index test
**65**	The Use of Machine Learning in MicroRNA Diagnostics: Current Perspectives	2022	10.2174/2211536611666220818145553	https://doi.org/10.2174/2211536611666220818145553	Wrong index test
**66**	Laboratory and Metabolomic Fingerprint in Heart Failure with Preserved Ejection Fraction: From Clinical Classification to Biomarker Signature	2023	10.3390/biom13010173	https://doi.org/10.3390/biom13010173	Wrong index test
**67**	Exploring the mechanisms of glycolytic genes involvement in pulmonary arterial hypertension through integrative bioinformatics analysis	2024	10.1111/jcmm.18447	https://doi.org/10.1111/jcmm.18447	Wrong index test
**68**	Identification of ARMCX3 as potential mitochondria-related biomarker for pulmonary arterial hypertension using integrated bioinformatics analysis and machine learning	2024	10.1161/circ.150.suppl_1.4144785	https://doi.org/10.1161/circ.150.suppl_1.4144785	Wrong index test
**69**	Identification of a Novel Diagnosis Model based on 5 Hub Genes for	2024	10.2174/0929867330666230605125512	https://doi.org/10.2174/0929867330666230605125512	Wrong index test

	Chronic Thromboembolic Pulmonary Hypertension				
**70**	microRNAs in congenital diaphragmatic hernia: insights into prenatal and perinatal biomarkers and altered molecular pathways	2024	10.1016/j.ajogmf.2024.101535	https://doi.org/10.1016/j.ajogmf.2024.101535	Wrong index test
**71**	Diagnostic MicroRNA Signatures to Support Classification of Pulmonary Hypertension	2025	10.1161/CIRCGEN.124.004862	https://doi.org/10.1161/CIRCGEN.124.004862	Wrong index test
**72**	Quantification of Arterial and Venous Morphologic Markers in Pulmonary Arterial Hypertension Using CT Imaging	2021	10.1016/j.chest.2021.06.069	https://doi.org/10.1016/j.chest.2021.06.069	Wrong index test output (segmentation only)
**73**	3D Visualization and Quantitative Assessment of the Pulmonary Arteries on CT Using Deep Learning Segmentation	2023	10.1161/circ.148.suppl_1.17842	https://doi.org/10.1161/circ.148.suppl_1.17842	Wrong index test output (quantification only)
**74**	AI-based Model for the Automatized Quantification and Prognosis of IPF Patients	2023	10.1164/ajrccm-conference.2023.D91	https://doi.org/10.1164/ajrccm-conference.2023.D91	Wrong index test output (quantification only)
**75**	Automatic Segmentation and Evaluation of Mitral Regurgitation Using Doppler Echocardiographic Images	2024	10.3390/bioengineering11111131	https://doi.org/10.3390/bioengineering11111131	Wrong index test output (quantification only)
**76**	External validation, radiological evaluation, and development of deep learning automatic lung segmentation in contrast-enhanced chest CT	2024	10.1007/s00330-023-10235-9	https://doi.org/10.1007/s00330-023-10235-9	Wrong index test output (quantification only)

**77**	Hierarchical Hybrid Networks for Automatic Pulmonary Blood Vessel Segmentation in Computed Tomography Images	2024	10.1109/TCBB.2023.3281828	https://doi.org/10.1109/TCBB.2023.3281828	Wrong index test output (quantification only)
**78**	Segmentation and Measurements of Major Blood Vessels from CT Pulmonary Angiography Using Deep Learning	2024	10.1101/2024.11.19.24317538	https://doi.org/10.1101/2024.11.19.24317538	Wrong index test output (quantification only)
**79**	Novel imaging techniques in pulmonary hypertension	2018	10.1097/HCO.0000000000000559	https://doi.org/10.1097/HCO.0000000000000559	Wrong index test (no ML/DL model)
**80**	Multivariable models for the diagnosis of pulmonary hypertension	2019	10.2459/JCM.0000000000000872	https://doi.org/10.2459/JCM.0000000000000872	Wrong index test (no ML/DL model)
**81**	Non-invasive Multimodality Cardiovascular Imaging of the Right Heart and Pulmonary Circulation in Pulmonary Hypertension	2019	10.3389/fcvm.2019.00024	https://doi.org/10.3389/fcvm.2019.00024	Wrong index test (no ML/DL model)
**82**	Pulmonary lung Doppler signals: normative data in a pediatric population compared with adults	2019	10.1007/s10877-019-00258-3	https://doi.org/10.1007/s10877-019-00258-3	Wrong index test (no ML/DL model)
**83**	The ‘great wait’ for diagnosis in pulmonary arterial hypertension	2020	10.1111/resp.13814	https://doi.org/10.1111/resp.13814	Wrong index test (no ML/DL model)
**84**	Predictive Modeling of Secondary Pulmonary Hypertension in Left Ventricular Diastolic Dysfunction	2021	10.3389/fphys.2021.666915	https://doi.org/10.3389/fphys.2021.666915	Wrong index test (no ML/DL model)
**85**	Pulmonary hypertension: Diagnosis and management	2021	10.3390/diagnostics11061066	https://doi.org/10.3390/diagnostics11061066	Wrong index test (no ML/DL model)
**86**	Finding Pulmonary Arterial Hypertension-	2022	10.1001/jamacardio.2022.0011	https://doi.org/10.1001/jamacardio.2022.0011	Wrong index test (no ML/DL model)

	Switching to Offense to Mitigate Disease Burden				
**87**	ESTABLISHMENT OF A NONINVASIVE DIAGNOSIS MODEL OF PORTOPULMONARY HYPERTENSION	2023	10.1097/HEP.0000000000000580	https://doi.org/10.1097/HEP.0000000000000580	Wrong index test (no ML/DL model)
**88**	Correlation analysis between lung ultrasound scores and pulmonary arterial systolic pressure in patients with acute heart failure admitted to the emergency intensive care unit	2024	10.1016/j.jointm.2023.08.005	https://doi.org/10.1016/j.jointm.2023.08.005	Wrong index test (no ML/DL model)
**89**	Development and validation of a Prediction Model for Chronic Thromboembolic Pulmonary Disease	2024	10.1186/s12931-024-03067-8	https://doi.org/10.1186/s12931-024-03067-8	Wrong index test (no ML/DL model)
**90**	Echocardiographic Screening Model for Improved Assessment of Atrial Septal Defect Closure: A Multicenter Retrospective Study	2024	10.1111/echo.70023	https://doi.org/10.1111/echo.70023	Wrong index test (no ML/DL model)
**91**	Improving Diagnosis and Management of Pulmonary Hypertension in Interstitial Lung Disease	2024	10.1016/j.chest.2024.06.3806	https://doi.org/10.1016/j.chest.2024.06.3806	Wrong index test (no ML/DL model)
**92**	Non-invasive estimation of pulmonary hypertension and clinical deterioration risk in pediatric congenital heart disease: Development and validation of predictive tools	2024	10.1097/CM9.0000000000003070	https://doi.org/10.1097/CM9.0000000000003070	Wrong index test (no ML/DL model)

**93**	Assessing left ventricular hemodynamic forces in systemic sclerosis patients: A pilot study	2025	10.1016/j.acvd.2024.10.118	https://doi.org/10.1016/j.acvd.2024.10.118	Wrong index test (no ML/DL model)
**94**	Utilization of artificial intelligence in echocardiography	2019	10.1253/circj.CJ-19-0420	https://doi.org/10.1253/circj.CJ-19-0420	Wrong population
**95**	Characterization of the Liver Parenchyma Using Parametric T1 and T2 Magnetic Resonance Relaxometry	2020	Not available	https://www.embase.com/search/results?subaction=viewrecord&id=LNCT04623528&from=export	Wrong population
**96**	Current Challenges and Recent Updates in Artificial Intelligence and Echocardiography	2020	10.1007/s12410-020-9529-x	https://doi.org/10.1007/s12410-020-9529-x	Wrong population
**97**	Clinical application of machine learning-based artificial intelligence in the diagnosis, prediction, and classification of cardiovascular diseases	2021	10.1253/circj.CJ-20-1121	https://doi.org/10.1253/circj.CJ-20-1121	Wrong population
**98**	Explainable artificial intelligence model for diagnosis of atrial fibrillation using holter electrocardiogram waveforms	2021	10.1536/ihj.21-094	https://doi.org/10.1536/ihj.21-094	Wrong population
**99**	Present Limitations of Artificial Intelligence in the Emergency Setting - Performance Study of a Commercial, Computer-Aided Detection Algorithm for Pulmonary Embolism	2021	10.1055/a-1515-2923	https://doi.org/10.1055/a-1515-2923	Wrong population
**100**	Diagnosis of COVID-19 with simultaneous accurate prediction of cardiac abnormalities	2023	10.1371/journal.pone.0290494	https://doi.org/10.1371/journal.pone.0290494	Wrong population

	from chest computed tomographic images				
**101**	Pneumothorax prediction using a foraging and hunting based ant colony optimizer assisted support vector machine	2023	10.1016/j.compbiomed.2023.106948	https://doi.org/10.1016/j.compbiomed.2023.106948	Wrong population
**102**	A machine learning approach to differentiate wide QRS tachycardia: distinguishing ventricular tachycardia from supraventricular tachycardia	2024	10.1007/s10840-024-01743-9	https://doi.org/10.1007/s10840-024-01743-9	Wrong population
**103**	Unlocking the potential of artificial intelligence in sports cardiology: does it have a role in evaluating athlete's heart?	2024	10.1093/eurjpc/zwae008	https://doi.org/10.1093/eurjpc/zwae008	Wrong population
**104**	Deep Learning-Based Identification of Echocardiographic Abnormalities From Electrocardiograms	2025	10.1016/j.jacasi.2024.10.012	https://doi.org/10.1016/j.jacasi.2024.10.012	Wrong population
**105**	Self-supervised learning for label-free segmentation in cardiac ultrasound	2025	10.1038/s41467-025-59451-5	https://doi.org/10.1038/s41467-025-59451-5	Wrong population
**106**	Artificial intelligence: practical primer for clinical research in cardiovascular disease	2019	10.1161/JAHA.119.012788	https://doi.org/10.1161/JAHA.119.012788	Wrong study design
**107**	Pulmonary hypertension in association with lung disease: Quantitative CT and artificial intelligence to the rescue? State-of-the-art review	2021	10.3390/diagnostics11040679	https://doi.org/10.3390/diagnostics11040679	Wrong study design

**108**	An expert opinion of the Polish Cardiac Society Working Group on Pulmonary Circulation on screening for chronic thromboembolic pulmonary hypertension patients after acute pulmonary embolism: Update	2022	10.33963/KP.a2022.0141	https://doi.org/10.33963/KP.a2022.0141	Wrong study design
**109**	A comprehensive review of applications of artificial intelligence in echocardiography	2024	10.1016/j.cpcardiol.2023.102250	https://doi.org/10.1016/j.cpcardiol.2023.102250	Wrong study design
**110**	AI-Enhanced ECG Applications in Cardiology: Comprehensive Insights from the Current Literature with a Focus on COVID-19 and Multiple Cardiovascular Conditions	2024	10.3390/diagnostics14171839	https://doi.org/10.3390/diagnostics14171839	Wrong study design
**111**	Artificial Intelligence Interpretation of the Electrocardiogram: A State-of-the-Art Review	2024	10.1007/s11886-024-02062-1	https://doi.org/10.1007/s11886-024-02062-1	Wrong study design (narrative review)
**112**	Deep learning supported echocardiogram analysis: A comprehensive review	2024	10.1016/j.artmed.2024.102866	https://doi.org/10.1016/j.artmed.2024.102866	Wrong study design (narrative review)
**113**	A Comprehensive Review of Artificial Intelligence (AI) Applications in Pulmonary Hypertension (PH)	2025	10.3390/medicina61010085	https://doi.org/10.3390/medicina61010085	Wrong study design (narrative review)
**114**	A systematic review of artificial intelligence tools for chronic pulmonary embolism on	2024	10.3389/fradi.2024.1335349	https://doi.org/10.3389/fradi.2024.1335349	Wrong study design (systematic review)

	CT pulmonary angiography				
**115**	Assessing the precision of machine learning for diagnosing pulmonary arterial hypertension: a systematic review and meta-analysis of diagnostic accuracy studies	2024	10.3389/fcvm.2024.1422327	https://doi.org/10.3389/fcvm.2024.1422327	Wrong study design (meta-analysis)
**116**	Explainable Artificial Intelligence in Radiological Cardiovascular Imaging—A Systematic Review	2025	10.3390/diagnostics15111399	https://doi.org/10.3390/diagnostics15111399	Wrong study design (systematic review)
**117**	Artificial intelligence to detect pulmonary hypertension in idiopathic pulmonary fibrosis: Study design	2023	10.1183/13993003.congress-2023.PA3494	https://doi.org/10.1183/13993003.congress-2023.PA3494	Wrong study design (protocol)
**118**	A Machine Learning Approach to Predict Pulmonary Hypertension in Newborns With Congenital Diaphragmatic Hernia: a Pilot Study	2020	Not available	https://www.embase.com/search/results?subaction=viewrecord&id=LNCT04609163&from=export	Trial registry record
**119**	Multimodality Imaging (Cardiovascular Magnetic Resonance Imaging, Echocardiography, and Nuclear Medicine Imaging) in the Screening, Diagnosis and Risk Stratification of Heart Failure With Preserved Ejection Fraction (HFpEF).	2020	Not available	https://www.embase.com/search/results?subaction=viewrecord&id=LNCT04603404&from=export	Trial registry record

**120**	Artificial Intelligence-Assisted Evaluation of Pulmonary Hypertension	2022	Not available	https://www.embase.com/search/results?subaction=viewrecord&id=LNCT05566002&from=export	Trial registry record
**121**	Characterization and Definition of Right Ventricular Injury in Patients Admitted to Intensive Care: Prospective Observational Study	2022	Not available	https://www.embase.com/search/results?subaction=viewrecord&id=LNCT05525936&from=export	Trial registry record
**122**	Applying Artificial Intelligence to the 12 Lead ECG for the Diagnosis of Pulmonary Hypertension: an Observational Study	2023	Not available	https://www.embase.com/search/results?subaction=viewrecord&id=LNCT05942859&from=export	Trial registry record
**123**	Deep Learning for Algorithmic Detection of Pulmonary Hypertension Using a Combined Digital Stethoscope and Single-lead Electrocardiogram	2023	Not available	https://www.embase.com/search/results?subaction=viewrecord&id=LNCT05873387&from=export	Trial registry record
**124**	Advanced Mutidimensional and Ultra High Resolution Computed Tomography to Inspect Cardiopulmonary Involvement in Progressive Fibrosing Interstitial Lung Diseases	2024	Not available	https://www.embase.com/search/results?subaction=viewrecord&id=LNCT06537934&from=export	Trial registry record
**125**	Exploration and Study on the Identification of Various Pulmonary Diseases Using Volatile Organic Compounds Biomarkers in Human Exhaled Breath	2024	Not available	https://www.embase.com/search/results?subaction=viewrecord&id=LNCT06528418&from=export	Trial registry record
**126**	Harnessing ECG Artificial Intelligence for	2024	Not available	https://www.embase.com/search/results?subaction=viewrecord&id=LNCT06462989&from=export	Trial registry record

	Rapid Treatment and Accurate Interpretation, an Open Label Randomized Controlled Trial				
**127**	Mechanisms of Pulmonary Vascular Dysfunction in Heart Failure	2024	Not available	https://www.embase.com/search/results?subaction=viewrecord&id=LNCT06331208&from=export	Trial registry record
**128**	Pediatric Ventricle Function Assessment Study	2024	Not available	https://www.embase.com/search/results?subaction=viewrecord&id=LNCT06739057&from=export	Trial registry record

A) Full-Text Exclusion Reasons — Mapping to PRISMA Categories

PRISMA Category (as reported in Figure 1)	Detailed Reason(s) Included	Count	% of Total
**Wrong Study Design**	Wrong publication type (n=11); Wrong study design (n=15); Trial registry record (n=11)	37	28.9%
**Wrong Outcomes**	Wrong outcome (n=11); Wrong reference standard (n=33)	44	34.4%
**No ML Model**	Wrong index test output (n=9); Wrong index test (n=25)	34	26.6%
**No Sufficient Data / Wrong Population**	Wrong population (n=13)	13	10.2%
**TOTAL**		**128**	**100%**

Supplementary Table S7. Per-study 2×2 counts and diagnostic accuracy for double-arm (comparative) studies, stratified by intended clinical task

Study	Modality	Validation type	Arm	TP	FP	FN	TN	Sensitivity	Specificity	Age (mean ± SD)
**PH detection (general)**										

**Imai 2024**	Chest X-ray	Internal — stratified split (60/40)	Traditional	25	7	5	64	0.833	0.901	51.9 ± 16.1
			ML/DL	28	1	2	70	0.933	0.986	62.5 ± 9.6
**Xie 2025**	CTPA	Single-center retrospective	Traditional	50	8	5	6	0.909	0.429	49.55 ± 16.94
			ML/DL	50	1	5	13	0.909	0.929	NA
**PH subtype differentiation**										
**Hirata 2024**	Echocardiography	External — independent testing dataset	Traditional	15	18	19	20	0.441	0.526	67 ± 14
			ML/DL	21	13	13	25	0.618	0.658	68 ± 15
**RHC-referral screening**										
**Argiento 2024**	Anamnestic + TTE	Internal — prospective validation cohort	Traditional	24	21	23	20	0.511	0.488	65.2 ± 14.8
			ML/DL	38	13	9	28	0.809	0.683	65.2 ± 13.6

Table S8: COMPARING PREVIOUS LITERATURE WITH CURRENT

Features	Dwivedi et al., 2024	Present Review
Review type	Systematic review and meta-analysis	Systematic review and meta-analysis
Clinical scope	Pulmonary arterial hypertension (PAH) only	Multiple pulmonary hypertension (PH) subtypes (WHO Groups 1–4) and neonatal PH
Index tests	AI/ML models based primarily on imaging	ML/DL models using echocardiography, ECG, chest radiography, CT, pulmonary function tests, and multimodal

		clinical data
Reference standard	Right heart catheterization (RHC)	RHC or validated echocardiographic criteria for neonatal PH
Number of included studies	Limited to studies available up to 2024	Nine studies published through October 2025
Comparative analysis of ML/DL vs conventional methods	Not performed	Yes; direct head-to-head comparison in comparative studies
Separate analysis by study design	No	Yes; non-comparative (single-arm) and comparative (double-arm) studies analysed separately
Primary statistical model	Conventional diagnostic meta-analysis methods	Reitsma hierarchical bivariate random-effects model applied consistently for all primary diagnostic accuracy outcomes
Diagnostic accuracy measures	Sensitivity and specificity	Sensitivity, specificity, PLR, NLR, DOR, PPV, NPV, SROC, and partial AUC
Evidence certainty assessment	Not reported	GRADE assessment performed independently for each analytical stratum
Risk of bias assessment	QUADAS-2	QUADAS-2
Novel contribution	Summarized AI performance in PAH	Broader PH population, direct comparison with conventional diagnostics, unified hierarchical diagnostic meta-analysis, and GRADE-based certainty assessment
